# Structure and assembly of pilotin-dependent and -independent secretins of the type II secretion system

**DOI:** 10.1371/journal.ppat.1007731

**Published:** 2019-05-13

**Authors:** S. Peter Howard, Leandro F. Estrozi, Quentin Bertrand, Carlos Contreras-Martel, Timothy Strozen, Viviana Job, Alexandre Martins, Daphna Fenel, Guy Schoehn, Andréa Dessen

**Affiliations:** 1 Dept. Biochemistry, Microbiology and Immunology, College of Medicine, University of Saskatchewan, Saskatoon, Saskatchewan, Canada; 2 Univ Grenoble Alpes, CNRS, CEA, Institut de Biologie Structurale (IBS), Grenoble, France; 3 Brazilian Biosciences National Laboratory (LNBio), CNPEM, Campinas, São Paulo, Brazil; Gifu University, JAPAN

## Abstract

The type II secretion system (T2SS) is a cell envelope-spanning macromolecular complex that is prevalent in Gram-negative bacterial species. It serves as the predominant virulence mechanism of many bacteria including those of the emerging human pathogens *Vibrio vulnificus* and *Aeromonas hydrophila*. The system is composed of a core set of highly conserved proteins that assemble an inner membrane platform, a periplasmic pseudopilus and an outer membrane complex termed the secretin. Localization and assembly of secretins in the outer membrane requires recognition of secretin monomers by two different partner systems: an inner membrane accessory complex or a highly sequence-diverse outer membrane lipoprotein, termed the pilotin. In this study, we addressed the question of differential secretin assembly mechanisms by using cryo-electron microscopy to determine the structures of the secretins from *A*. *hydrophila* (pilotin-independent ExeD) and *V*. *vulnificus* (pilotin-dependent EpsD). These structures, at approximately 3.5 Å resolution, reveal pentadecameric stoichiometries and C-terminal regions that carry a signature motif in the case of a pilotin-dependent assembly mechanism. We solved the crystal structure of the *V*. *vulnificus* EpsS pilotin and confirmed the importance of the signature motif for pilotin-dependent secretin assembly by performing modelling with the C-terminus of EpsD. We also show that secretin assembly is essential for membrane integrity and toxin secretion in *V*. *vulnificus* and establish that EpsD requires the coordinated activity of both the accessory complex EpsAB and the pilotin EpsS for full assembly and T2SS function. In contrast, mutation of the region of the S-domain that is normally the site of pilotin interactions has little effect on assembly or function of the ExeD secretin. Since secretins are essential outer membrane channels present in a variety of secretion systems, these results provide a structural and functional basis for understanding the key assembly steps for different members of this vast pore-forming family of proteins.

## Author summary

*Vibrio vulnificus* and *Aeromonas hydrophila* are two bacterial species that are emerging as significant human pathogens. The major virulence mechanism utilized by these species is the Type II secretion system (T2SS) that functions in exporting toxic proteins produced by the bacterium into the infected individual. The T2SS is a large complex of proteins that form a molecular piston-like structure and a pore on the surface of the cell (called the secretin) that together use energy to transport toxic proteins out of the bacterial cell. In this study we used cryo-electron microscopy to characterize the structure of the secretins from *V*. *vulnificus* and *A*. *hydrophila* and in addition determined the crystal structure of EpsS from *V*. *vulnificus*. We present a functional analysis of proteins EpsAB and EpsS that serve to enable and promote assembly of the secretin pore in the outer membrane of *V*. *vulnificus*. These results identify *V*. *vulnificus* as a species whose assembly of the T2SS secretin involves the activity of two different systems, notably the peptidoglycan-modulating complex EpsAB and the outer membrane lipoprotein pilotin EpsS. In contrast, mutational analysis of the C-terminal region of ExeD at which pilotins normally interact provides further evidence that the previously identified ExeAB complex is the only factor required for secretin assembly in *A*. *hydrophila*.

## Introduction

Pathogenic bacteria commonly employ the Type II Secretion System (T2SS) for the release of protein toxins. In many cases these proteins are fundamentally responsible for the pathologies associated with infection including attachment, invasion and defense against host responses [1]. *Vibrio vulnificus* is a food-borne pathogen that causes a rapidly progressing and frequently fatal septicemia, and it employs the T2SS to secrete different toxic virulence factors including hemolysins, chitinases, proteases, lipases, and phospholipases [2]. The opportunistic pathogen *Aeromonas hydrophila*, which is a causative agent of skin and soft tissue infections as well as gastrointestinal tract infections, also utilizes the T2SS for the secretion of a wide variety of degradative and toxic proteins, including proteases, lipases, amylases, enterotoxins and the well-studied pore-forming toxin aerolysin [3]. For these reasons, the assembly, structure and function of the T2SS is an important focus of research on the pathogenesis of these and many other bacterial infections.

The T2SS is comprised of 12–16 proteins, generically termed GspC-O, GspAB, and GspS (with alternative species-specific nomenclatures), with the latter three components being variably present in different bacteria [4]. The system includes an inner membrane platform of GspC-E-F-L-M, a periplasmic pseudopilus composed of GspG-H-I-J-K and the outer membrane secretion channel GspD, called the secretin. Homologs of the highly conserved secretin are also found in other secretion and surface assembly systems including the Type III Secretion system (T3SS) and the Type IV pilus assembly system (T4P), the latter of which shares a common ancestry with the T2SS [5–11]. Notably, the secretin and the assembly platform are thought to assemble independently and transiently, and there is only a single, recent example where an intact T2SS has been purified and characterized in a stable form [12].

In numerous species the targeting, assembly and stability of the T2SS secretin in the outer membrane require the function of a small lipoprotein that binds to its C-terminal region (the S-domain) and serves to pilot secretin monomers from the inner to the outer membrane via the LOL lipoprotein sorting pathway [13–15]. In the well-studied *Klebsiella oxytoca* and *Dickeya dadantii* T2SS systems, the pilotins PulS and OutS are respectively responsible for piloting the secretin to the outer membrane, and their loss results in localization and in some cases assembly of the secretin in the inner membrane [16]. Peptides harboring the sequences of the C-terminal helix of the S-domain have been shown to bind to pilotins of different systems through NMR, crystallography, and other biochemical and cellular techniques; in some cases, they were shown to be unstructured in solution but to become stabilized upon binding to the target pilotin [9, 17–20]. The T2SSs of enterotoxigenic (ETEC) and enteropathogenic *E*. *coli* (EPEC) species have also been shown to express a T2SS pilotin (GspS_β_/AspS) and it shares the same general biochemical features of the PulS/OutS family (lipoproteins of approx. 120 amino acids) but exhibits little if any conservation in sequence [21, 22] or 3D-structure with PulS/OutS [22]. Consistent with the two families of pilotins identified in the T2SS of gamma-proteobacteria, there are two distinct families of secretins with which they interact. The PulS/OutS pilotins recognize the secretins of the Gsp_α_ or *Klebsiella*-type, and the GspS_β_/AspS pilotins recognize the secretins of the Gsp_β_ or *Vibrio*-type [21, 22].

**Fig 1:**
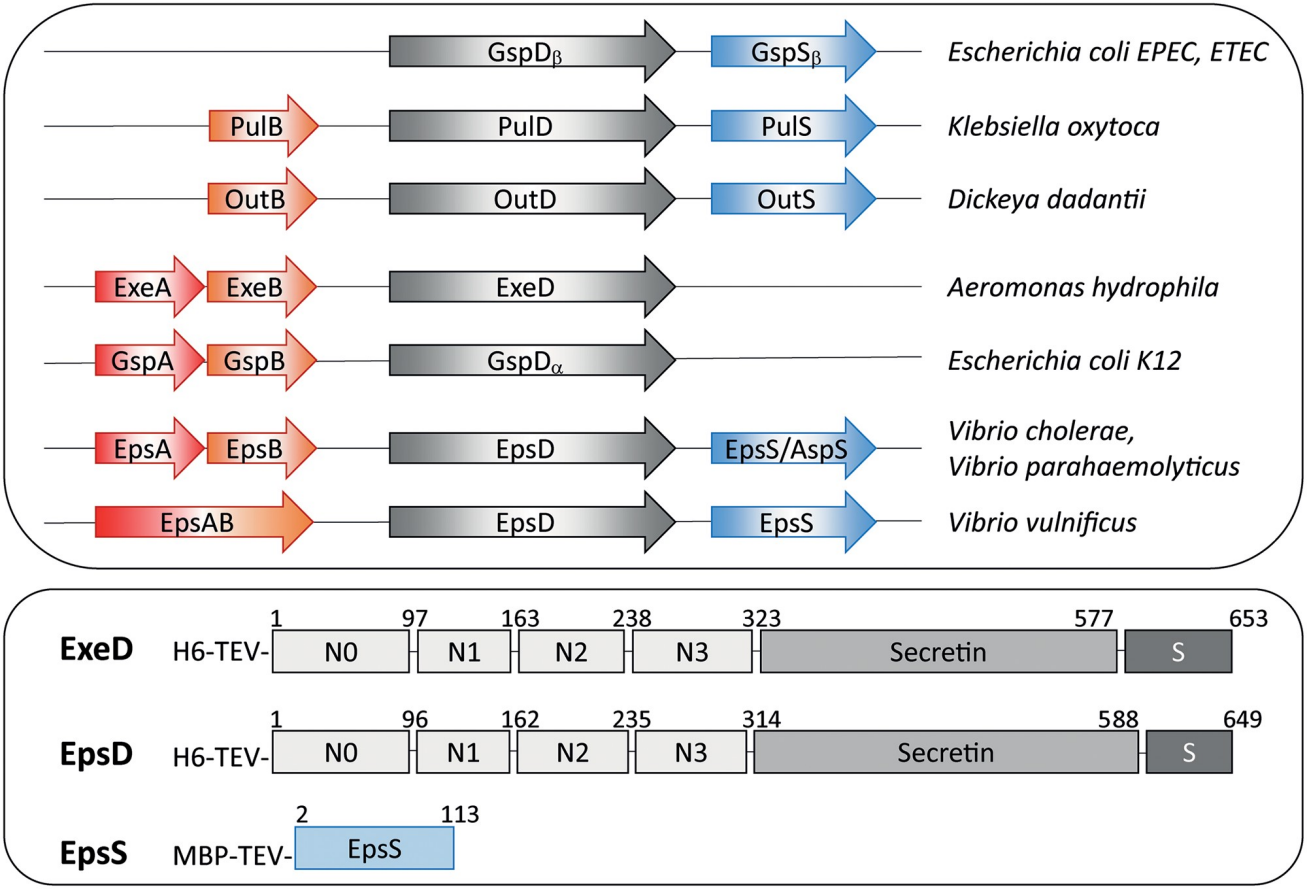
Presence of AB accessory proteins and pilotins in various bacteria and constructs employed to express *A*. *hydrophila* ExeD, *V*. *vulnificus* EpsD and the *V*. *vulnificus* pilotin EpsS. (A) The accessory proteins, secretins and pilotins identified in the T2SS of the indicated bacteria are shown. (B) Structures of fusion proteins used to purify ExeD, EpsD and EpsS. Numbers refer to the residues of the native secretins and pilotin after signal sequence processing. S: S-domain; H6: His-tag; MBP: Maltose-Binding Protein; TEV: cleavage site for TEV protease.

In *Aeromonas hydrophila*, the Exe T2SS is encoded by two operons, *exeC-N* and *exeAB* (Fig 1) [23]. A GspS pilotin homolog is not found in the bacterium, and piloting and assembly of the ExeD secretin are dependent instead on the inner membrane complex ExeAB; in its absence, ExeD monomers accumulate in the inner membrane [24]. ExeA can be cross-linked to peptidoglycan *in vivo*, and genetic and structural analyses identified the location and critically important residues of its peptidoglycan-binding site [25–27]. Furthermore, the purified ExeA periplasmic domain forms large multimers in association with peptidoglycan *in vitro* [25]. ExeD monomers may be recruited to the assembly site created by ExeAB through interactions with the ExeB component of the complex, which was shown to bind to the N0/N1 subdomains of ExeD [28]. These results suggest that ExeAB is involved in organizing the assembly of the secretin in association with both peptidoglycan and the outer membrane.

Taken together, the available evidence presented above suggests that in PulS/OutS and GspS_β_/AspS-containing systems the LOL lipoprotein pathway is used to target and transfer secretins to the outer membrane, whereas in GspAB-containing systems an alternative route of transfer and assembly which involves interactions with peptidoglycan is used. However, it is also possible that in some bacteria, both of these assembly pathways operate simultaneously in secretin assembly. In *D*. *dadantii* the ExeB homolog OutB is required for efficient secretion and was further shown to both stabilize and to cross-link to OutD [29, 30]. GspAB homologs have been identified as EpsA and EpsB in *V*. *cholerae* and *V*. *parahaemolyticus* and as an EpsAB fusion in *V*. *vulnificus* [31] (Fig 1). In contrast to the effect of *exeA* mutations in *Aeromonas spp*., in all three of the *Vibrio* species secretin assembly was significantly decreased but not abolished in *epsA* and *epsAB* mutants, suggesting the presence of another route to secretin assembly in these bacteria. A potential explanation for this discrepancy was provided by the discovery of the GspS_β_/AspS pilotin, a homolog of which is present in the *Vibrios*; however, the relative roles of the two pathways in *Vibrio* secretin assembly have not been examined.

In order to gain insight into secretin assembly via both the GspAB and the pilotin-dependent pathways, we employed a multi-technical approach and used cell-free synthesis, cryo-EM, X-ray crystallography, and bacterial genetics. First, we determined the cryo-EM structure of *A*. *hydrophila* ExeD as the paradigm of a secretin that is assembled through the actions of the ExeAB accessory complex. In addition, we also solved the cryo-EM structure of the EpsD secretin of *V*. *vulnificus*, which encodes both an EpsAB fusion and a GspS_β_/AspS pilotin (EpsS). In order to understand pilotin recognition of EpsD, we solved the high resolution crystal structure of EpsS, determined its importance for secretin assembly in *V*. *vulnificus*, and generated a model for the EpsD:EpsS complex. In light of recently determined structures of other secretins, these results yield significant insight into the evolutionary relationship between secretin structures and modes of assembly.

## Results and discussion

### Isolation and cryo-EM analyses of EpsD and ExeD secretins

**Fig 2:**
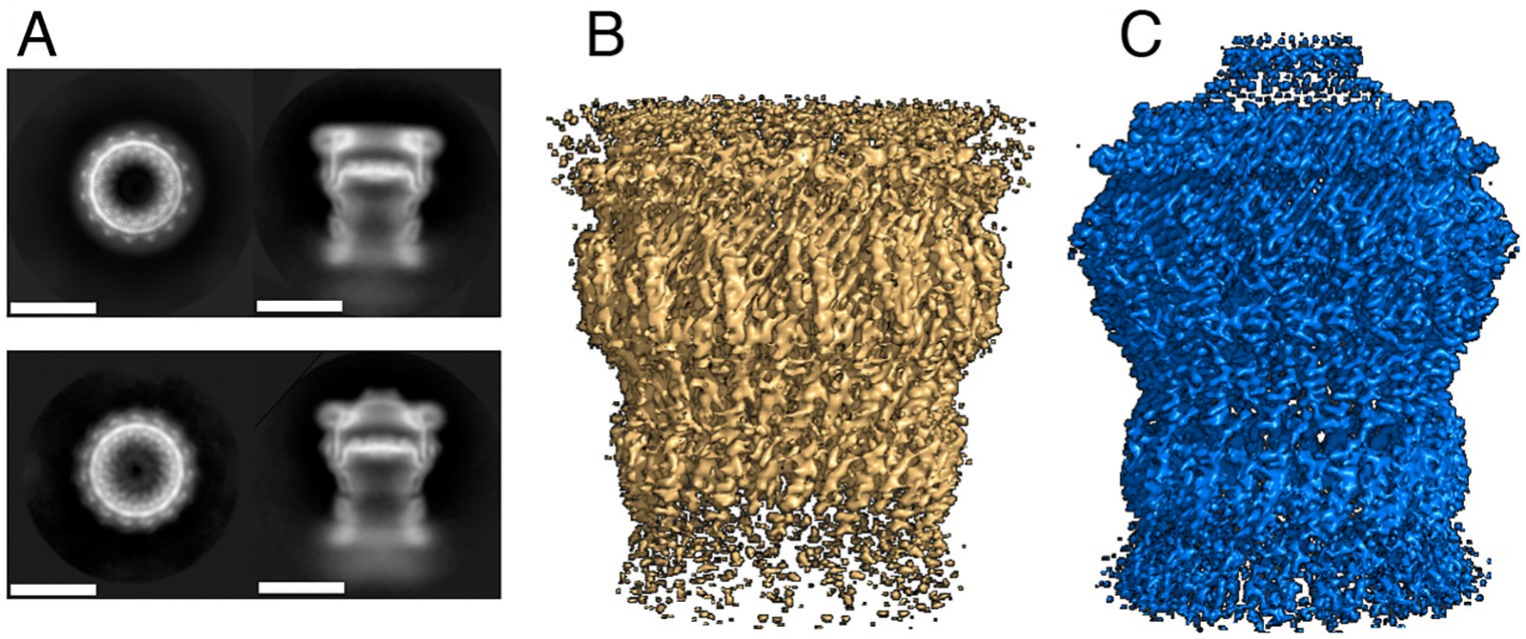
**2D class averages of side and top views** of (A) ExeD (upper panel) and EpsD (scale bars 100 Å). (B) 3D reconstructions of ExeD and EpsD (C), contoured at level 5 with Pymol 2.2.0.

The full-length forms of EpsD and ExeD were produced in a cell-free system [6] in the presence of liposomes, extracted as intact multimers, and purified to homogeneity in Zwittergent 3–14 (Zw3-14). SDS-PAGE and negative stain EM analyses of both EpsD and ExeD showed that they remained multimeric after purification (Fig 2 and S1 Fig). The two complexes were subsequently frozen on Quantifoil holey carbon grids covered by a thin layer of continuous amorphous carbon. This extra carbon layer was required in order to increase the number of side views (in its absence almost only top views were observed; S2 Fig). The grids were imaged using a Polara (FEI) electron microscope working at 300 kV and movies were recorded using a direct electron detector camera. For each sample, 2D image classification (Fig 2) was performed using Relion [32]. Cryo-EM data collection and refinement statistics can be found in S1 Table.

**Fig 3:**
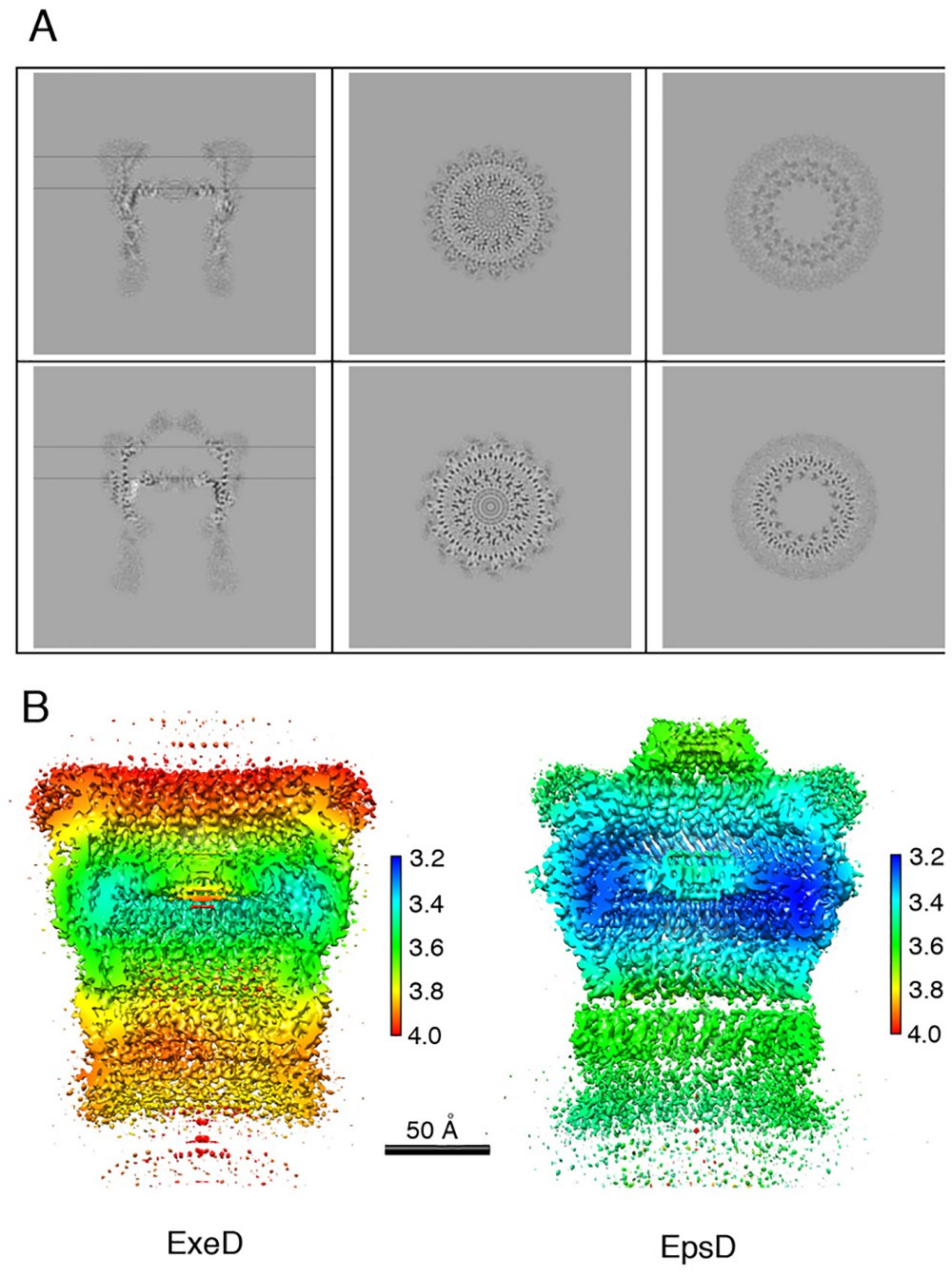
ExeD and EpsD are pentadecamers. (A) Cross-sections of the secretin maps along (left) and across the symmetry axis (center and right), of ExeD (top panels) and EpsD (bottom panels). The cross-sections across the symmetry axes are at the heights indicated by the horizontal lines. (B) Local resolution of the density maps of ExeD and EpsD, colored according to resolution (in Å) estimated by ResMap [67].

Analyses of top views of class averages (Fig 2, S2 and S3 Figs) revealed that, for both secretins, symmetry is unambiguously pentadecameric (C15 symmetry) (Fig 3). Despite the fact that other secretins have also been purified with C12, C13, and C16 symmetries [5, 6, 33–37], the presence of 15 unequivocal densities in the class average images pointed to the stability of this pentadecameric arrangement, as also seen in structures of other intact secretins or assembled secretion systems [9–11, 38]. The final reconstructions included 46,126 and 51,960 particles for EpsD and ExeD, respectively. The resolutions of the resulting symmetrized maps were estimated to be in the range of 3.4 Å for EpsD and 3.7 Å for ExeD by employing the gold standard FSC criterion (Fig 3 and S4 Fig). Maps were traced with the aid of models 5WQ8 and 5WQ7 corresponding to the recently published structures of EpsD from *V*. *cholerae* and GspD from *E*. *coli* K-12 [11], with which our molecules display 83% and 55% sequence identity, respectively.

### Arrangement and individual domains of ExeD and EpsD

**Fig 4:**
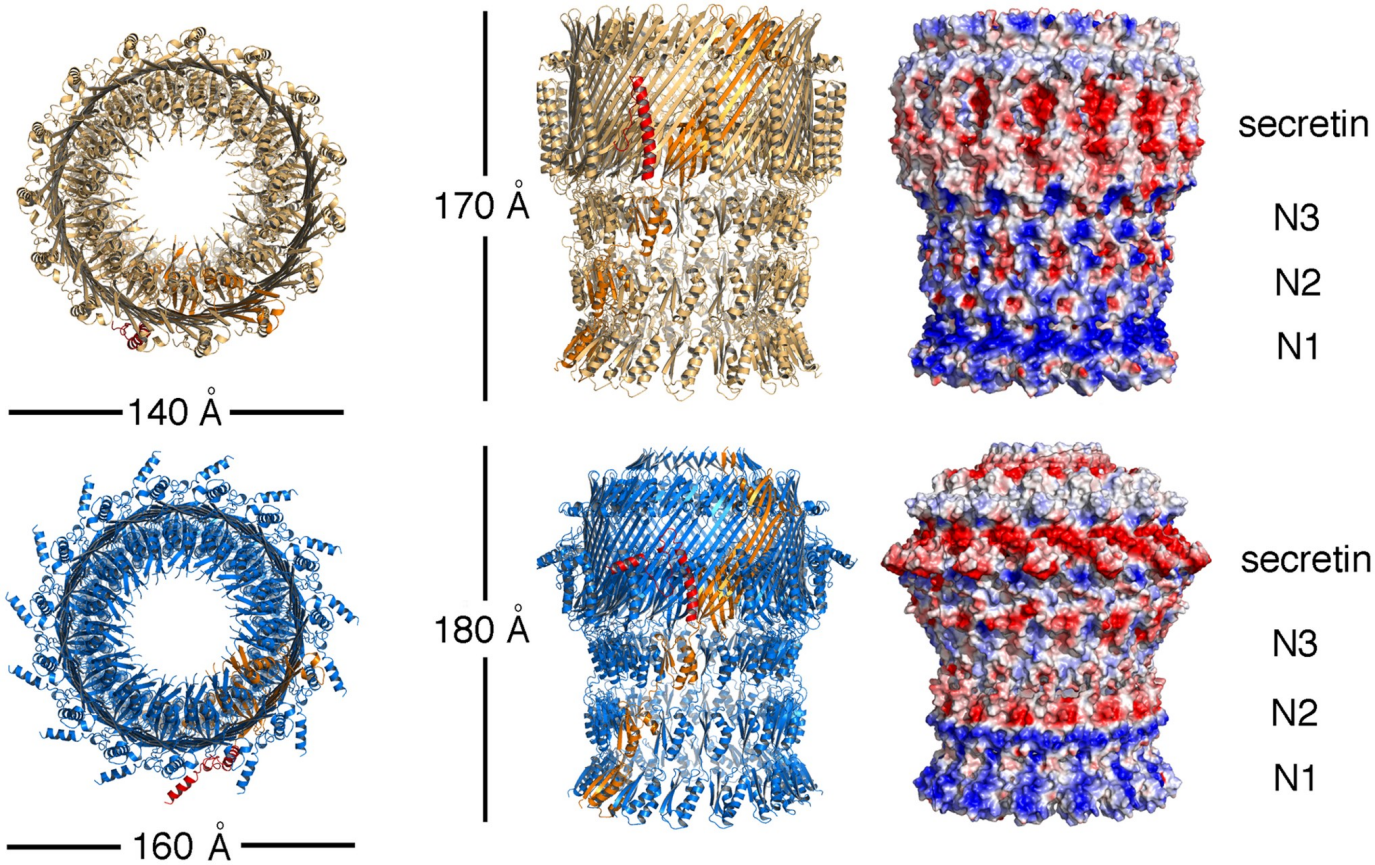
Models of ExeD (top) and EpsD (bottom). Fifteen monomers form each one of the structures, and in both cases one monomer is colored in orange. The S-domain of the same monomer is indicated in red. (Right) Surface potentials of ExeD and EpsD, where blue and red colors represent basic and acidic residues, respectively. The membrane-binding region, in addition to a hydrophobic belt (white), also displays an acidic region formed mostly by residues from the S domain. In both cases, due to high flexibility in the N-terminal region, fitting was guided by the GspD models [11].

**Fig 5:**
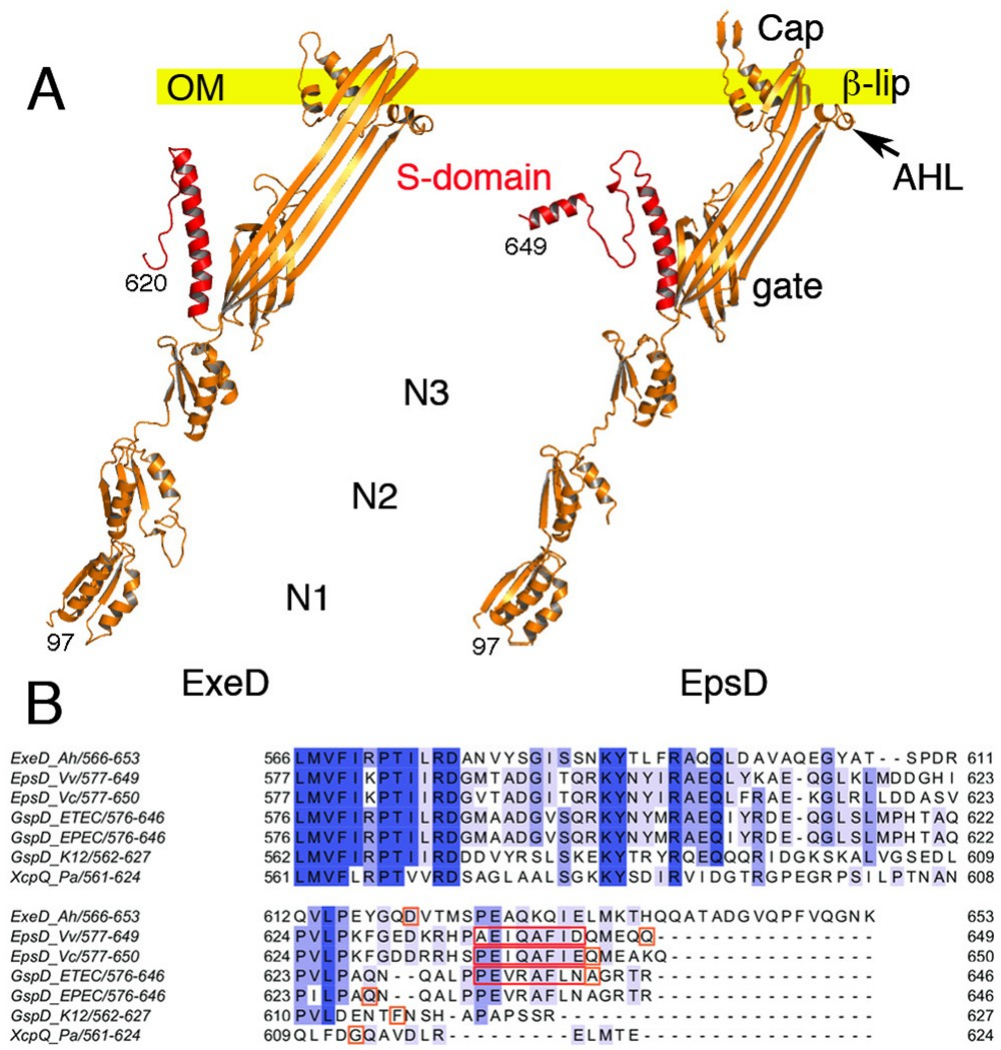
Domain arrangements of the single monomers of ExeD and EpsD and alignment of secretin S-domains. (A) Single monomers of ExeD and EpsD are shown as they would be oriented to the plane of the outer membrane. The S-domains are indicated in red. The outer membrane (OM) is indicated as a yellow bar. In both secretin structures, domain N1 was mostly modeled from X-ray crystal structures. (B) Multiple sequence alignment of secretin S-domains from species *V*. *cholerae* (5WQ8), *V*. *vulnificus* (this work), *A*. *hydrophila* (this work), *E*. *coli* ETEC (5ZDH), *E*. *coli* EPEC (5W68), *E*. *coli* K-12 (5WQ7) and *P*. *aeruginosa* (5WLN). Amino acids that comprise α-12 are boxed in red. The last residue observed in each cryo-EM structure is boxed in orange.

In both structures, the protomers mostly fold into linear associations of individually folded N-terminal α/β domains (N1, N2, N3) interlinked by flexible linkers, and a C-terminal region whose notable characteristic is the ‘secretin domain’. In each protomer, this domain is formed by the stacked 4-stranded β-sheet, which forms the outer barrel, as well as a smaller sheet with shorter strands, forming the inner barrel (Figs 4 and 5). This double barrel feature has been noted in the structure of a number of secretins [9, 11] and the 60-stranded antiparallel architecture that is generated has been suggested as being linked to the notable stability of these molecules.

Despite the fact that the above-mentioned resolutions allowed for unequivocal domain assignment, local resolution analyses indicated variance in some regions (Fig 3b). The N3 and ‘secretin domain’ of both EpsD and ExeD displayed the highest resolutions and were traced unequivocally through analysis of the maps. In both structures, however, the periplasmic N0-N2 domains were relatively flexible, as in the case of other cryo-EM secretin structures, and tracing required close modeling starting from the structures of GspD from *E*. *coli* and *V*. *cholerae* [11]. Interestingly, recent crystallographic analyses of the N0—N2 domains of the *Pseudomonas aeruginosa* GspD homologue XcpQ revealed a hexamer of dimers, and further indicated pronounced conformational flexibility in this region of the secretin [39]. In both ExeD and EpsD structures, however, most residues within the N-terminal chamber ‘gate’ could be assigned (with the exception of the interconnecting loops (Fig 4), indicating that both structures are in ‘closed conformation’).

One of the clearest differences between the two types of secretins lies in the outermost region that is positioned towards the exterior of the cell. In EpsD this region is covered by a ‘cap’, formed by 15 aligned β-hairpins that lie on top of the membrane-embedded region (Fig 4). This region points towards the inside of the pore, closing the structure on the top. In ExeD, the sequence corresponding to the β-hairpin is absent and the structure is finalized by the β-lip (Fig 5), a common feature seen in both structures. The β-lip is the major membrane-spanning region, and in the pentadecamer its associated 4-stranded β-sheets form a short ‘hydrophobic belt’ [10, 40]. In both ExeD and EpsD, the β-lip is juxtaposed to an amphipathic helical loop (the AHL) that has also been observed in all secretins that have been structurally characterized to date. This highly conserved sequence is thought to play a role in initiating contact between secretin monomers and the inner leaflet of the bilayer, with association through its polar side allowing an increase in effective monomer concentration [9, 40]. An analysis of the surface potential of both ExeD and EpsD indicates that only the top rim of the molecules, which corresponds to the β-lip and the AHL, has a clear hydrophobic character (white in Fig 4, right), suggesting that it is only this small area that is introduced into the outer membrane with the rest of the molecule located in the periplasm, as also observed in the structure of other secretins [9, 10].

### The S-domain, but not α-12, is present in both pilotin-dependent and independent secretins

As mentioned above, the inner membrane ExeAB complex is both necessary and sufficient for the assembly of ExeD; in accordance with this, the *Aeromonads* do not have known pilotins. However, sequence comparisons reveal that ExeD does contain a recognizable S-domain including residues that could potentially correspond to helix α-12, a fully solvent-exposed, 8-residue helix that has been reported to bind pilotins in analogous regions in other secretins [17, 18, 39, 41, 42] (red boxes in Fig 5b). Interestingly, pilotin-dependent secretins carry a conserved Ala-Phe sequence within α-12, whereas those that do not have known pilotins display either a shorter C-terminus or an unrelated sequence in this region (Fig 5b). In our ExeD sequence, for example, the conserved Ala-Phe motif is absent and is replaced by Lys-Gln, residues whose high polarity would not be able to play the same roles of the two hydrophobic amino acids of the motif. In addition, in our cryo-EM structure of ExeD, the last visible residue in the electron density map is Asp620. This indicates that the C-terminal tail, which includes the region discussed above, is flexible and not traceable and most probably does not fold into a stable domain.

*V*. *vulnificus* EpsD, however, displays a well folded S-domain (residues 588–649) with a clear α-12 (Figs 4 and 5). The Ala-Phe motif is present in EpsD, much as in its counterparts from *V*. *cholerae* and ETEC [11, 41]. Thus, the presence of this motif within the S-domain could be a signature of pilotin dependence for secretin assembly, a suggestion that is presently applicable to secretins whose cryo-EM structures have been solved. Pilotins have been divided into two distinct classes, PulS/OutD- and GspS_β_/AspS-like, and high-resolution structures of members of each family have revealed that the folds are distinct [22, 43]. We thus sought to explore the requirement of the α-12 Ala-Phe motif of EpsD for pilotin recognition by solving the high-resolution crystal structure of EpsS (the pilotin from *V*. *vulnificus*), modeling its interaction with the S-domain of EpsD, and verifying its importance in EpsD assembly *in cellulo*.

### Interactions between EpsS and the S domain of EpsD in atomic detail

Crystals of EpsS from *V*. *vulnificus* (residues 2–113) diffracted to 1.75 Å at the ESRF synchrotron in Grenoble. The structure was solved by molecular replacement with Arcimboldo Schredder [44] with the structure of AspS from *V*. *cholerae* (49% identity) being employed as a model [22]. ARP/wARP [45] was used to rebuild the model and reduce bias from the original molecular replacement solution. EpsS harbors 1 molecule per asymmetric unit, and data collection and refinement statistics are included in S2 Table.

**Fig 6:**
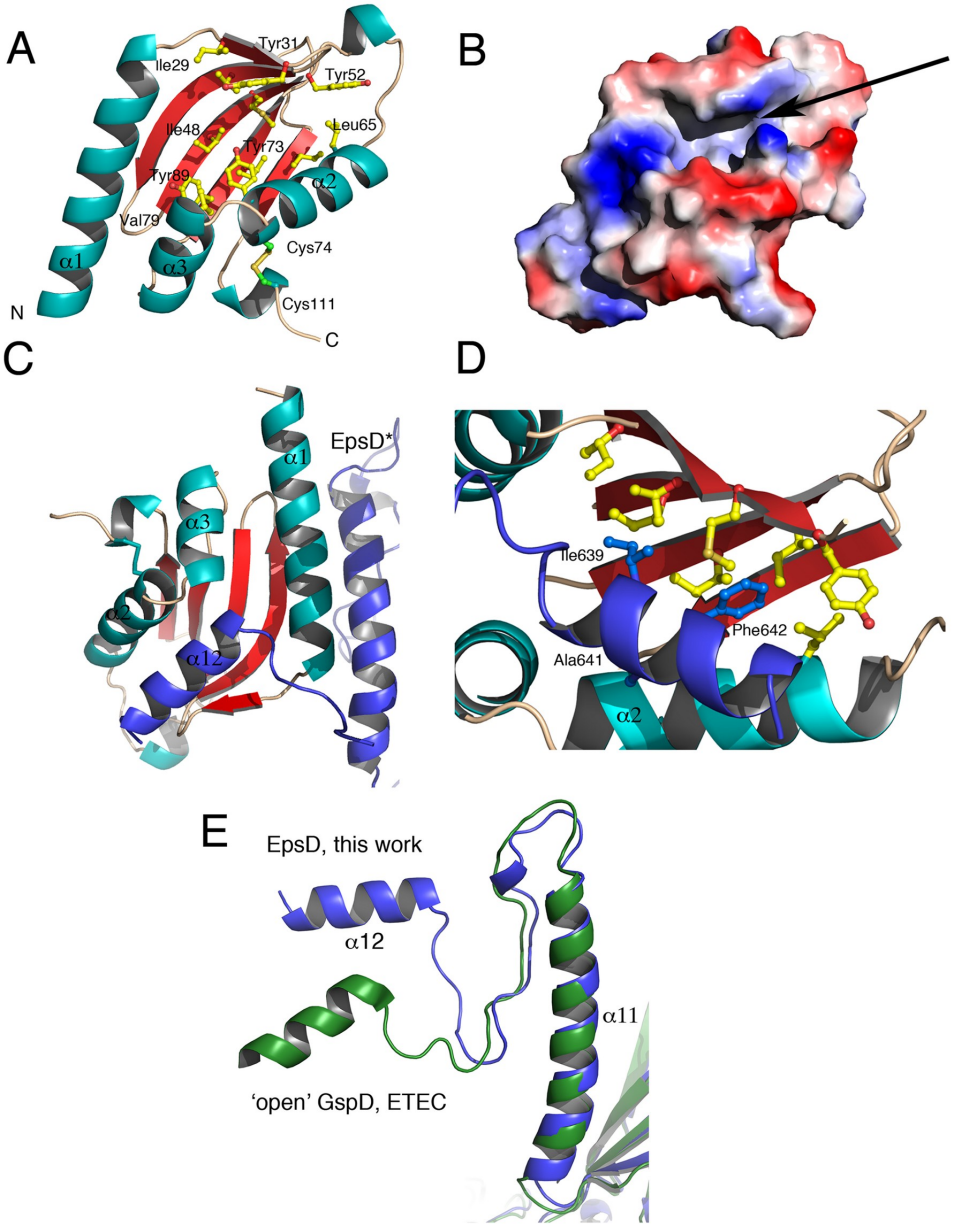
Structure and surface diagram of EpsS from *V*. *vulnificus*. (A) EpsS is a compact molecule consisting of a 5-stranded antiparallel sheet buttressed by four major helices. The central sheet displays a highly hydrophobic character. (B) A surface diagram of EpsS suggests that there is a tunnel-like region formed at the center of the molecule, indicated with an arrow, that represents the binding site for α-12 from EpsD. (C) The central crevice of EpsS contacts α-12 of EpsD in its ‘pilotin-recognizing’ conformation. Details of modeling can be found in the text. EpsD* is in dark blue. (D) A close-up of the EpsS-EpsD* interaction indicates that the Hyd-X-Ala-Phe motif of EpsD*’s α-12 fits snugly into the hydrophobic patch on EpsS’ central sheet, indicating why this is a conserved signature for these pilotin-recognizing secretins. (E) Comparison of the S-domains of EpsD from *V*. *vulnificus* and GspD from ETEC in its ‘open’, pilotin-bound state [41]. Since pilotin recognition requires ‘opening’ of α-12, in order to understand how EpsS recognizes EpsD we first generated an EpsD model where α-12 was modeled onto the ‘open’ position of the GspD C-terminal helix.

EpsS is a compact molecule composed of a central, 5-stranded β-sheet and 4 flanking α-helices, and is reminiscent to that of AspS from *V*. *cholerae* (RMSD 0.715 Å) [22]. In EpsS, an elongated cavity, whose walls are formed by the α-helices, is located at the center of the molecule. The floor of the cavity, formed by the central β-sheet, is decorated with hydrophobic resides (Ile48, Tyr73, Tyr89, Ile91), and a disulfide bond generated between Cys74 and Cys111 stabilizes the cavity wall formed by α-2 and α-4 (Fig 6a and 6b).

During the preparation of this manuscript, a paper reporting the cryo-EM structure of GspD from Enterotoxigenic *E*. *coli* (ETEC) bound to its pilotin was reported [41]. In that structure, despite the clarity of the localization of the pilotin complexed to the S-domain of GspD, the low local resolution (4.0–5.0 Å) did not allow for a clear tracing of all loop regions nor the description of the detailed interactions with the S-domain. Thus, in order to gain insight into the structural determinants of EpsS/EpsD recognition in atomic detail, we set out to model our 1.75 Å structure of EpsS onto our 3.4 Å cryo-EM map of EpsD. Interestingly, as in the case of GspD [11], α-12 from the S-domain of EpsD was in a conformation that did not allow for EpsS binding, and our attempts to fit EpsS into this region led to clear clashes. We thus based our modeling studies on the cryo-EM GspD:AspS structure [41]. Yin and co-workers showed that, in the case of GspD, in order to allow for pilotin binding, the S-domain must undergo a conformational modification that exposes the C-terminal helix (Fig 6e). Thus, in order to generate a form of EpsD that mimicked the ‘open’ form of GspD, we overlaid our EpsD structure onto the cryo-EM model of GspD (the one in complex with AspS), manually adjusted our C-terminal helix to overlay onto that of GspD, and used this new, open model (that we called EpsD*) to perform manual docking of our EpsS crystal structure.

EpsS employs the central part of its β-sheet to interact with α-12 of EpsD* (Fig 6c and 6d). These residues include Leu65 and Tyr73 from α-2, as well as Tyr89, Ile91, and Ile93 from the β-sheet of the pilotin. However, we also observed that residues located in β-1 of EpsS as well as the loops that connect the individual strands in the central β-sheet can clash with amino acids lining EpsD*’s α-12 if the pilotin is to remain in this conformation in the complex. This suggests that, upon complex formation, not only must α-12 from the secretin move away from the central β-barrel, but the pilotin itself must also slightly open its central tunnel in order to accommodate residues from α-12. This hypothesis is supported by the observation that in the structure of the GspD:AspS complex from ETEC, β-1 and the interconnecting β-sheet loops of AspS were not traceable (S5 Fig) [41], indicating flexibility of these residues of the pilotin in the bound state. Notably, in the structure of the apo form of AspS, these same residues ‘close’ over the tunnel region, also indicating flexibility in this area [22].

The EpsD*:EpsS complex fits in such a way that it allows the hydrophobic residues Ile639, Ala641, and Phe642 from EpsD*’s α-12 to be placed within EpsS’s pocket (Fig 6d). While Ala641 and Phe642 correspond to the strictly conserved motif mentioned above, Ile639 corresponds to a Val in GspD from ETEC and EPEC, thus representing a conserved mutation (Fig 5b). Thus, this observation confirms that the presence of the Hyd-X-Ala-Phe motif, where Hyd is an apolar residue, within the S-domain of a secretin is a telltale sign of pilotin dependence. Notably, mutation of the corresponding Phe residue in α-12 from ETEC GspD abrogated pilotin binding in a pulldown assay, lending further support to this idea [41]. In order to further explore these findings, we examined assembly and function of wild-type and mutated forms of the Eps T2SS from *V*. *vulnificus in cellulo*.

### Both the pilotin EpsS and the accessory protein EpsAB are involved in *V*. *vulnificus* secretin assembly

**Fig 7:**
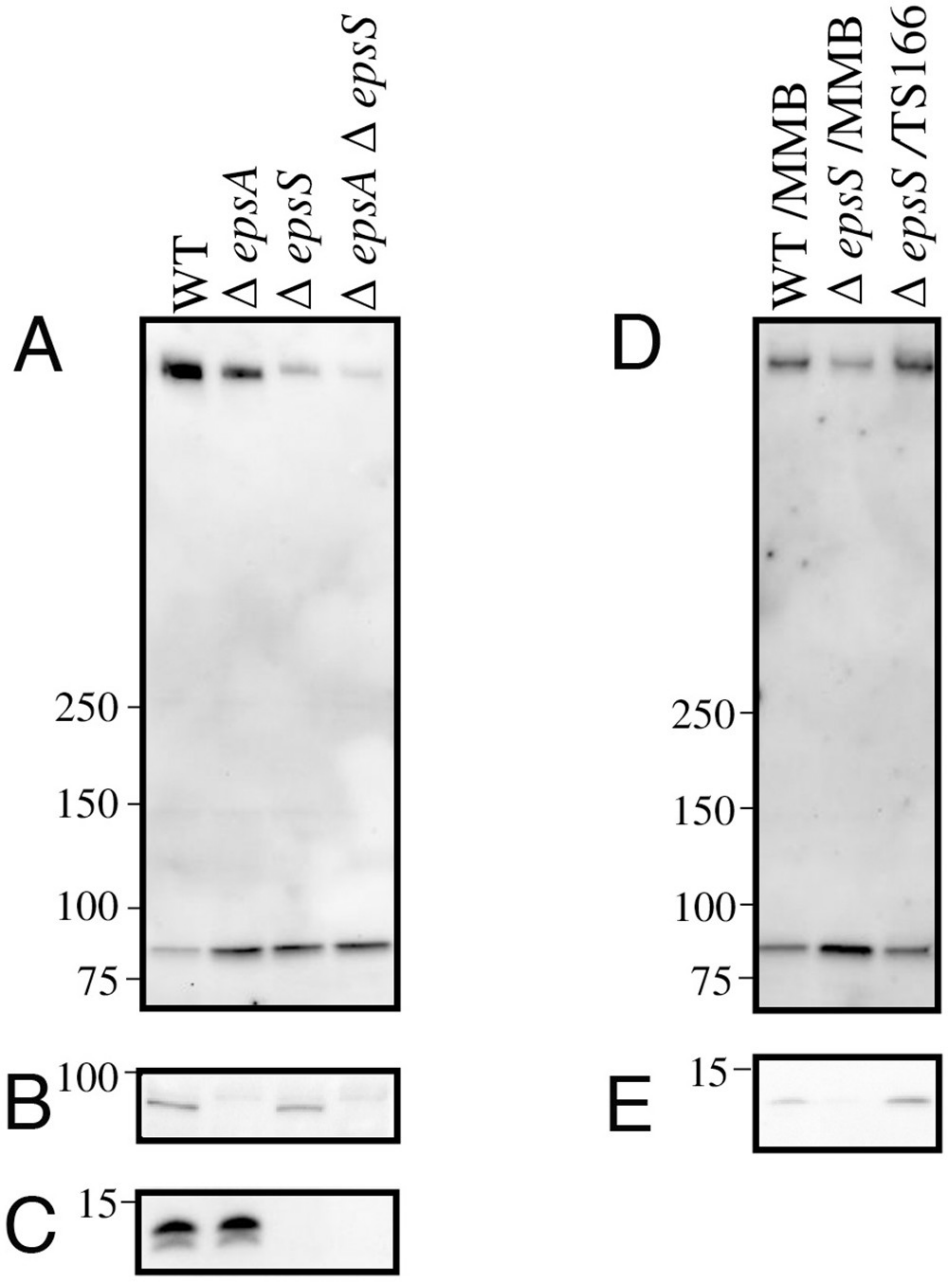
The effect of *epsA* and *epsS* mutations on EpsD secretin assembly are additive. Immunoblot analysis of whole cell samples of WT, Δ*epsA*, Δ*epsS* and Δ*epsA*Δ*epsS* strains of *V*. *vulnificus* with (A) anti-EpsD (B) anti-EpsA and (C) anti-EpsS antibodies. Whole cell samples of WT and *epsS* strains containing empty vector (MMB) together with the *epsS* strain containing TS166 (expressing EpsS *in trans*) were analyzed by immunoblot with (D) anti-EpsD and (E) anti-EpsS antibody.

**Fig 8:**
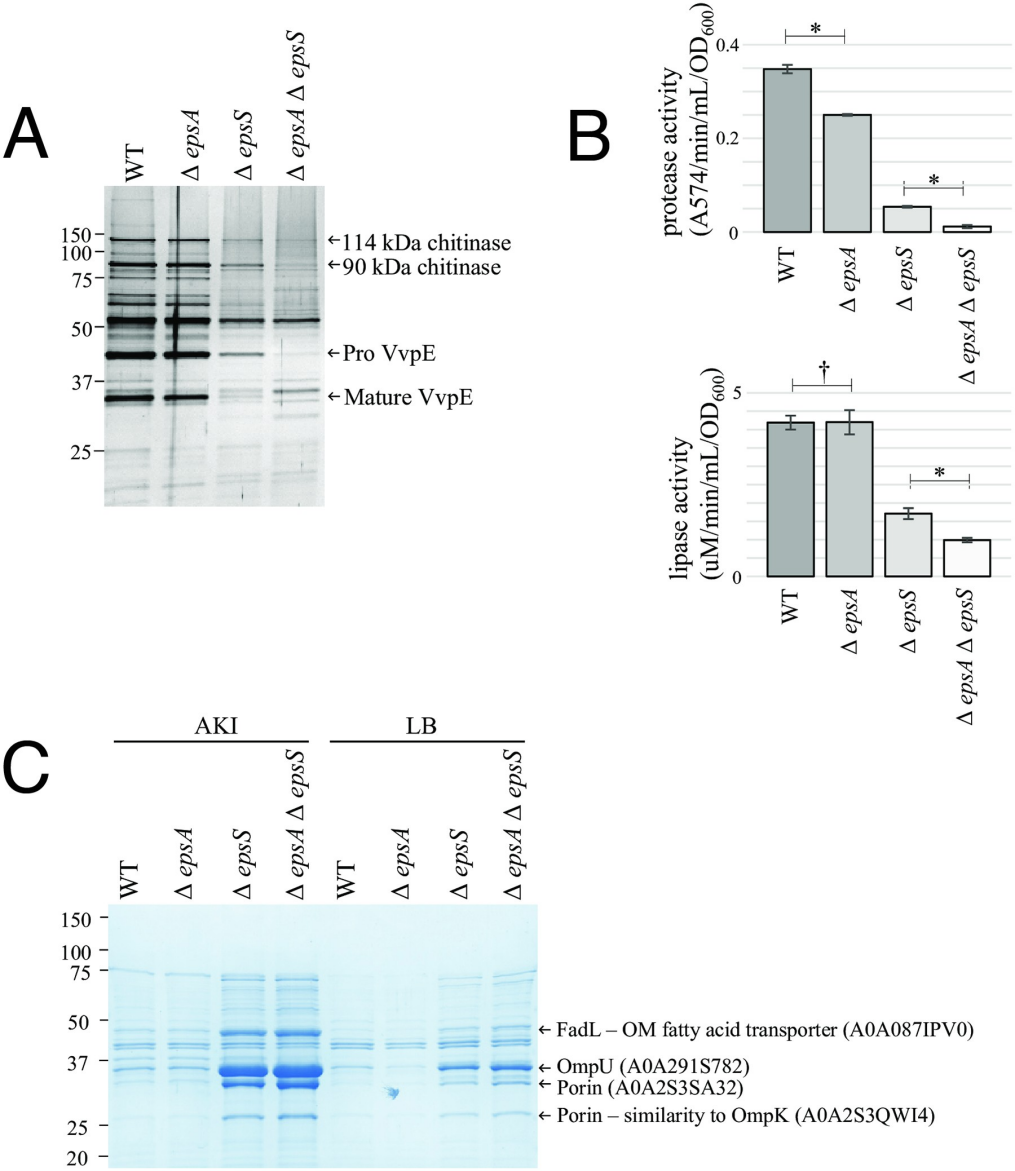
The effect of *epsA* and *epsS* mutations on T2SS function are additive. (A) All secreted proteins (silver stained gel) and (B) protease and lipase activities released by *V*. *vulnificus* WT, Δ*epsA*, Δ*epsS* and Δ*epsA*Δ*epsS* strains. Arrows identify abundant proteins which are present in progressively lower amounts in culture supernatants. (C) Increased vesicle release by mutants defective in assembly of the EpsD secretin. Each lane contains an equal volume of membrane fragments concentrated 25-fold by ultracentrifugation of AKI or LB culture supernatants. Protein bands designated by arrows were identified by MS. Statistical analyses used a two-sided unpaired T-test (†, non-significant; *, significant [P<0.005]).

Previous work had described the partial requirement for the EpsAB accessory complex in assembly of the EpsD secretin in *Vibrio* species *cholerae*, *vulnificus* and *parahaemolyticus* [31]. To determine if the EpsS pilotin was also required for secretin assembly in *Vibrio* species and the relative requirement for EpsA and EpsS in secretin assembly and function of the T2SS, *V*. *vulnificus* deletion mutants of *epsA*, *epsS* and both *epsA* and *epsS* were created. The amount of secretin assembled in the mutants and secretion of T2SS substrates was then assessed in comparison to that of the wild-type strain. As shown in Fig 7a, individual deletions of *epsA*, *epsS* and both *epsA* and *epsS* conferred an additive deleterious effect on the amount of EpsD assembled that was accompanied by an increase in the amount of unassembled EpsD monomer observed. The defect in secretin assembly in the Δ*epsS* strain could be complemented by expression of EpsS *in trans* (Fig 7d). Likewise, Δ*epsA*, Δ*epsS* and Δ*epsAΔepsS* had an additive negative effect on the secretion of most of the extracellular proteins produced by the wild-type, as observed by SDS-PAGE of culture supernatants (Fig 8). Mass spectrometry identified four of the most highly abundant of these secreted proteins as two chitinases (114 kDa and 90 kDa) and the proprotein and mature versions of the hemagglutinin/protease VvpE (Fig 8a) [46], while enzyme assays demonstrated the progressive reduction in lipase and protease activities (Fig 8b) produced by the single and double mutants.

Outer membrane instability is characterized by the leakage of periplasmic proteins out of the cell and increased vesiculation of outer membrane material. Previous studies of the effects of complete loss of secretin assembly in *V*. *cholerae* demonstrated impaired integrity of the outer membrane in T2SS mutants that are unable to synthesize the secretin [47]. Similar effects were also observed in T2SS mutants of *V*. *vulnificus*, and were attributed to intra-periplasmic accumulation of the autolysin protease VvpS [48]. Examination of the relative amounts of outer membrane vesicle release into the supernatant of wild-type, Δ*epsA*, Δ*epsS* and Δ*epsAΔepsS* cultures revealed that an inverse correlation exists between assembly of the secretin and vesicle release (Fig 8c). That is, a step-wise increase in outer membrane vesicular material (confirmed by mass spectroscopic detection of three porins and an outer membrane fatty acid transporter, FadL) was observed in Δ*epsA*, Δ*epsS* and Δ*epsΔepsS* mutants in comparison to wild-type, suggesting a greater impairment in secretin assembly and T2SS function in these strains. These results are a further indication of a progressive inability to assemble the secretin in the *epsA* and *epsS* single and double mutants. Together, these results demonstrate that EpsS functions as the pilotin of the EpsD secretin in *Vibrio* species, and also show that the *V*. *vulnificus* T2SS utilizes the activities of both the accessory complex (EpsAB) and the pilotin (EpsS) for secretin assembly.

In contrast, in *A*. *hydrophila* all available evidence has suggested that only the ExeAB complex is required for secretin assembly and that it interacts with the amino-terminal region of ExeD rather than the S-domain during the assembly process. This further raises the question, amenable to genetic analysis in the *A*. *hydrophila* T2SS, of what if any role the S-domain plays in the assembly and/or stability of this pilotin-independent secretin.

### The *A*. *hydrophila* ExeD secretin shows little dependence on the α-12 helix region or further C-terminal residues of its S-domain for assembly and function

**Fig 9:**
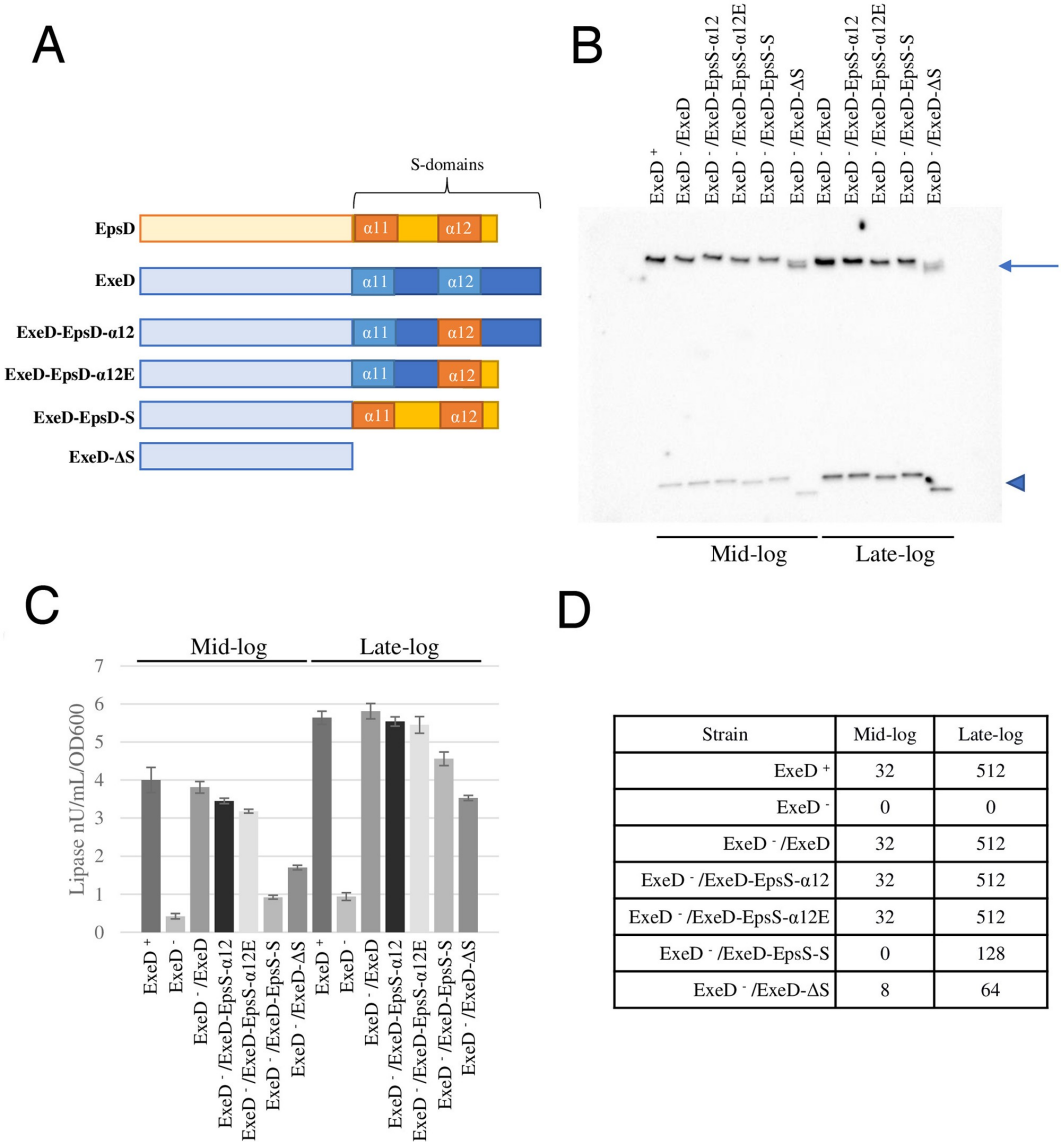
Secretin assembly and secretion by *A*. *hydrophila* strains expressing ExeD variants. (A) Scheme of WT and mutant secretins used in this study. (B) Secretin immunoblot of whole cell samples of Ah65 or AhD14 expressing WT or S-domain mutant ExeDs grown to mid- and late-log phase. The assembled secretin and monomeric ExeD are shown by an arrow and triangle, respectively. (C) Lipase activities in culture supernatants of Ah65 or AhD14 expressing WT or S-domain mutant ExeDs. (D) Aerolysin titres of culture supernatants.

In order to address the possible specific role of the α-12 helix region and the rest of the S-domain in the assembly, localization and stability of ExeD, four mutants were constructed in which part or all of the S-domain-encoding region of *epsD* was exchanged into the equivalent regions of *exeD* (Fig 9a). In the first of these (ExeD-EpsD-α-12) the α-12 sequence of EpsD was exchanged into ExeD, while in the second (ExeD-EpsD-α-12E) the α-12 sequence of EpsD was exchanged into ExeD and the last 19 amino acids of the ExeD S-domain were removed and replaced with EQQ, which corresponds to the C-terminus of EpsD. In a third mutant (ExeD-EpsD-S) the entire S-domain of ExeD, including the α-11 helix, was replaced by that of EpsD, and in the fourth mutant the entire S-domain of ExeD was deleted (ExeD-ΔS).

**Fig 10:**
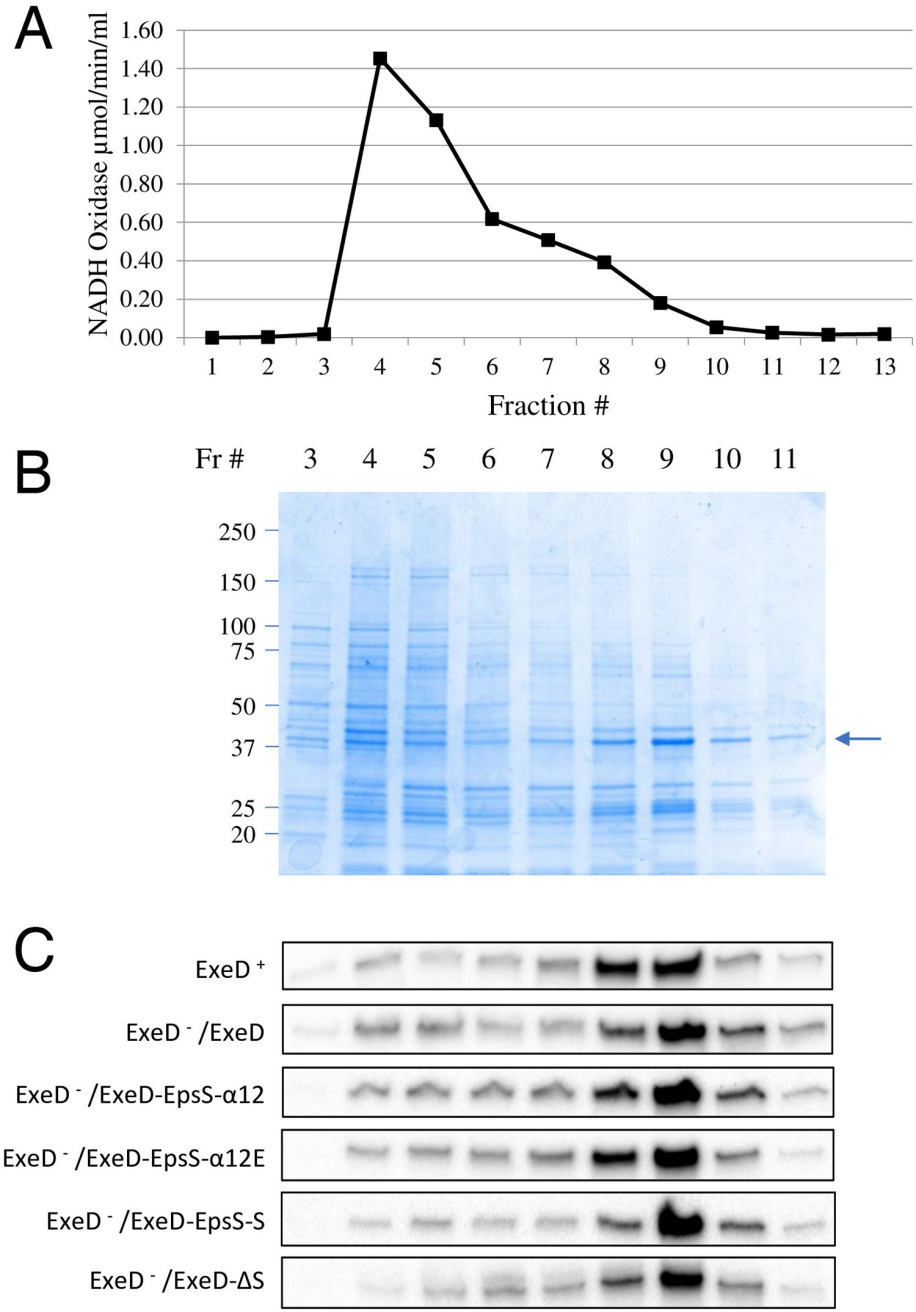
Membrane localization of WT and S-domain mutant ExeD secretins. Inner and outer membranes of WT Ah65 and *exeD*^-^ mutant AhD14 expressing either WT or S-domain mutant ExeDs were fractionated by isopycnic sucrose-density-gradient ultracentrifugation. (A) The activity of the inner membrane marker NADH oxidase across a representative gradient from AhD14/pVACD (ExeD^-^ /ExeD) is shown. (B) The SDS-PAGE protein profile across the same gradient, identifying fractions with peak outer membrane porin intensities (arrow). (C) Secretin immunoblot analyses of the membrane fractions from Ah65 or AhD14 expressing WT or S-domain mutant ExeDs.

The mutant *exeD*s were constructed in plasmid pVACD, and the variants were then assayed for their ability to be assembled and to complement the secretion defect in the *exeD*^-^ deletion strain AhD14 [24]. Cells were induced for expression and grown to mid- and late-log phase in culture. As shown in Fig 9b, each of the three exchange-mutant ExeDs could be assembled into the secretin, although the ExeD-EpsD-α-12E and ExeD-EpsD-S variants were produced in lower amounts or were less stable during synthesis and/or translocation, particularly after growth to higher density. The ExeD-ΔS variant was stably produced at a substantially lower level, and the assembled form migrated as two closely spaced but distinct forms on SDS-PAGE electrophoresis. It should be noted that all of the wild-type and mutant cell samples (and membrane samples, *cf*. Fig 10c) were prepared for electrophoresis by heating to 95°C in sample buffer containing 2% SDS, indicating that once assembled, the secretin variants including the ExeD-ΔS mutant were essentially as stable as was the wild-type form.

AhD14 cells expressing wild-type ExeD and each of the four variants were also compared with respect to the secretion of lipase and aerolysin as a measure of function of the complete Exe T2SS, again at the mid- and late- log growth phases. In the lipase assay it was clear that the ExeD-EpsD-α-12 and ExeD-EpsD-α-12E variants were as proficient in secretion as was the wild-type throughout the growth cycle (Fig 9c). In contrast, the ExeD-EpsD-S variant-expressing strain substantially lagged the other strains in secretion during the mid-log phase of growth, although by the late-log growth phase it had caught up to the wild-type and the ExeD-EpsD-α-12 and ExeD-EpsD-α-12E variants. The ExeD-ΔS variant also lagged in lipase secretion during mid-log phase growth, but it too had partially caught up by late-log phase, secreting approximately 60% of the lipase secreted by the wild-type. Similar results were observed for the secretion of the hemolysin aerolysin, assayed using a semi-quantitative endpoint titre assay of mouse erythrocyte hemolysis (Fig 9d). The wild-type and the ExeD-EpsD-α-12 and ExeD-EpsD-α-12E variants secreted equivalent amounts of the toxin at both growth phases assayed, while the ExeD-EpsD-S and ExeD-ΔS mutants secreted no aerolysin and a small amount, respectively, in the mid-log growth phase, and greater (but not wild-type) levels by the late-log growth phase.

For the ExeD-EpsD-S and ExeD-ΔS -expressing strains in particular, it was possible that the reduced lipase and aerolysin secretion profiles, despite substantial secretin assembly, were caused by inappropriate assembly in the inner rather than in the outer membrane. In order to examine this possibility, isopycnic sucrose-density-gradient ultracentrifugation was used to fractionate the inner and outer membranes of the wild-type and mutant strains. Fig 10a and 10b show the NADH oxidase and SDS-PAGE protein profiles of the gradient used to separate the membranes of AhD14 expressing wild-type ExeD from pVACD (ExeD^-^ /ExeD), demonstrating successful separation of the two membranes. Similar fractionations with equivalent results were performed for the *A*. *hydrophila* strain Ah65 and each of the strains expressing the mutant ExeDs. The membrane fractions were then electrophoresed and immunoblotted with ExeD anti-serum, as shown in Fig 10c. The results indicate that for wild-type and mutant strains, the large majority of the secretin present co-fractionated with the porin used as the marker for the outer membrane vesicle fractions.

The effects of the *exeD* S-domain mutations studied here stand in stark contrast to those that would be expected for a typical pilotin-dependent secretin such as PulD, OutD, or GspD [4]. Many studies have shown that for each of these secretins, interaction with the corresponding pilotin is a critical requirement for the correct localization and assembly of the secretin and the function of the T2SS it belongs to. The lack of such a requirement for ExeD assembly thus allowed genetic analysis of the requirement for the regions of its S-domain that would otherwise not be possible. The essentially normal assembly and function of the ExeD-EpsD-α-12 variant indicates that the specific residues of the α-12 region do not play a strong role in the structure of ExeD secretin. Although the ExeD-EpsD-α12E variant was stably produced in lower amounts, it too appeared normal in function, suggesting that the C terminal 19 amino acids are also not critically important to ExeD structure or function in the assembled state. In contrast, however, the ExeD-EpsD-S variant, in which the EpsD α11 helix-containing region was also exchanged into ExeD, was both assembled in lower amounts and significantly compromised in function once assembled, suggesting that the interactions between this helix and the neighbouring monomer that are evident in the cryo-EM structure (Fig 4) play an important role in both the assembly and the function of the secretin. Finally, and surprisingly, although the ExeD variant completely lacking the S-domain was very poorly assembled and even more severely compromised in function, its partial assembly into a typically temperature- and detergent-resistant multimeric structure indicates that the S-domain does not play a fundamental role in the structural stability of the secretin once it has assembled.

### Models for secretin assembly in pilotin-dependent and–independent secretion systems

Since the T2SS is a macromolecular machine utilized by Gram-negative species to survive and thrive in a wide range of environments, the complement of substrates secreted by different species is diverse and highlights how they have altered the system to meet their own requirements. Specialization of the T2SS has resulted in alterations of the core set of proteins that comprise the bulk of the system, and has also led to the evolution of additional proteins involved in secretin assembly that include EpsA, EpsB and EpsS. Although EpsAB is an inner membrane complex that binds peptidoglycan and EpsS is an outer membrane lipoprotein, both proteins could perform the same function by drastically different mechanisms. Recent evidence suggests that the secretin likely forms upon the spontaneous assembly of secretin monomers and insertion in their outer membrane by a β-barrel assembly machinery (BAM)-independent mechanism that is initiated once an effective concentration of EpsD monomers are localized near the inner leaflet [10]. EpsAB and EpsS may localize EpsD monomers via interactions with the N-terminal N0-N1 domain and C-terminal S-domains, respectively, effectively performing the same function to localize and concentrate secretin monomers in the cell envelope to induce multimerization and insertion of the secretin in the outer membrane.

If EpsAB and EpsS perform analogous functions in localizing and concentrating the secretin monomer in the cell envelope, this would not explain why some species such as the *Vibrios* encode both the accessory factors and a pilotin. The reason may lie in when and/or where during cell growth the T2SS is assembled. If assembly of the T2SS is coordinated with cell division at the divisome, then assembly of the large megadalton-sized secretin in the outer membrane could bypass the need for remodelling of the peptidoglycan (PG) meshwork, a situation previously described for assembly of the Type IVa pilus in *Pseudomonas aeruginosa* [49]. In this way, EpsS could be required and sufficient for localization and concentration of EpsD monomers at the divisome. If the T2SS is assembled when the cell is not dividing or in a region that contains mature PG, a PG-remodelling function of EpsAB could be integral for reorganization of PG and localization and concentration of the EpsD monomer. In both cases, integration of the secretin in the inner leaflet of the outer membrane could be mediated primarily by the Amphipathic Helical Loop (AHL) of EpsD (in the proximity of the β-lip, highlighted in Fig 5). Interestingly, the AHL is the most highly conserved region of EpsD among species regardless of the presence or absence of a pilotin, and has previously been suggested to be involved in the initial steps in assembly of the secretin into the outer membrane [9, 11].

The possibility that accessory proteins EpsAB and EpsS effectively function to concentrate the EpsD monomer, leading to spontaneous assembly of the secretin and insertion in the outer membrane, would explain why it has been shown in several species including *A*. *hydrophila* [24] that overexpression of the secretin monomer can complement the lack of accessory proteins or pilotins that are crucial for assembly of the secretin at wild-type levels of expression. Thus, it is plausible that the lipoprotein pilotins and the PG-interacting EpsAB complex represent distinct mechanisms that Gram-negative bacteria have evolved to localize and concentrate the T2SS secretins and that these mechanisms could operate independently or together in different bacteria. The specific interactions between pilotins and the conserved Ala-Phe α-12 motif, highlighted here, could provide a strategy that could facilitate differentiation of the specific mechanism to be employed.

## Materials and methods

### Expression and purification of ExeD, EpsD and EpsS

The EpsD and ExeD secretins were synthesized *in vitro* as described using the cell-free expression system at the ISBG Cell Free Platform (IBS Grenoble) [6]. The *A*. *hydrophila exeD* and *V*. *vulnificus epsD* genes without the region encoding their signal sequences were amplified utilizing primer pairs AhD24TF/AhD24TR and VvD24TF/VvD24TR respectively and cloned using *BamHI* and *XhoI* into pIVEX2.4T (modified from pIVEX2.4a (5 Prime)) which added a TEV cleavage site linker and His-6 tag to the N terminus of the proteins synthesized. The cell free synthesis reactions were conducted at a plasmid concentration of 16 μg/ml in an extract volume of 9 ml and incubated ON at 30°C. Liposomes prepared as previously described [6] utilizing an *E*. *coli* total lipid extract (Avanti) were added to the reactions at a final concentration of 1 mg/ml to promote multimerization of the secretins. Following synthesis, the reaction mixture was centrifuged at 18,000 X g for 45 minutes to recover the liposomes and associated secretin. For EpsD, after resuspension in 20 mM Tris-HCl, 150 mM NaCl pH 7.5 (TN buffer) the liposome/secretin preparation was solubilized with 2% Zw3-14 in TN for 1 hr at RT, centrifuged at 12,000 X g for 10 min and the supernatant was diluted to 0.2% Zw3-14 in TN containing 40 mM Imidazole. The sample was applied to a Ni-NTA column equilibrated in the same buffer. Following a 10-column volume (CV) wash with the starting buffer, the column was eluted with a 10 CV gradient of TN containing 0.5 M imidazole and 0.2% Zw3-14. Pooled fractions containing the secretin were then concentrated by centrifugation using a 100 kDa cutoff membrane and applied to a Superose 6 column (GE Healthcare) equilibrated in TN containing 0.1% Zw3-14. The multimer secretin peaks were collected and used for cryo-EM analysis. For ExeD, the same procedure was followed except that following solubilization of the liposome/secretin preparation, the sample was diluted and fractionated on a Ni-NTA column in buffer containing 0.5% Zw 3–14 and applied onto a Superose 6 column in TN buffer containing 0.4% Zw 3–14.

The *V*. *vulnificus epsS* gene without the region encoding its signal sequence and the N-terminal cysteine of the lipid-modified form was amplified using oligonucleotides EpsSTEVF and EpsSTEVR and cloned using *EcoRI* and *NcoI* into vector pMBP-parallel1 [50] which generated a *malE-epsS* fusion gene with a TEV protease cleavage site at the fusion joint. A *BglII-SalI* fragment of this clone was then exchanged into vector pMAL-p4X (NEB), resulting in a fusion gene encoding MBP-EpsS which contains the MBP signal sequence for translocation of the fusion protein to the periplasm and a TEV cleavage site between MBP and EpsS.

For expression and purification of EpsS, the p4MBP-EpsS clone was transformed into *E*. *coli* BL21 cells, and transformants grown at 30 °C with shaking in LB medium containing 100 μg/ml ampicillin. At an OD_600_ of approximately 0.8, the culture was induced with 0.4 mM IPTG and further incubated for 4 hours. The culture was then centrifuged at 12,000 X g for 10 min and the cells resuspended in 10 mM NaPO_4_, 400 mM NaCl pH 7.5 followed by breakage by 3 passages through a cell disruptor operating at 1000 PSI. The ruptured cells were then centrifuged at 50,000 X g for 30 min and the supernatant applied to a 5 ml MBP-trap column (GE) equilibrated with the same buffer. Following a 10 CV wash of the column, the MBP-EpsS fusion was eluted with a gradient to 1 mM Maltose in the starting buffer. Pooled peak fractions from this elution were digested for 4 h at RT with a 1/50 mass ratio of His-tagged TEV, which was then removed by passage of the extract through a Ni-NTA column equilibrated in PBS. The digested MBP-EpsS was then re-applied to the MBP-trap column to remove MBP. The flow through was concentrated and applied to a Superdex 75 column (GE Healthcare) equilibrated in TN. Peak fractions from this column were pooled, re-concentrated to 10 mg/ml and used for crystallization trials and optimization.

### Cryo-EM

Samples were frozen with a Vitrobot Mark IV (FEI) at 100% humidity and 2 seconds of blotting time, force 1. Holey carbon grids (QUANTIFOIL) were used with an extra continuous 2 nm thick layer of amorphous carbon in order to have more particles presenting side-views. Without the extra carbon layer, we observed almost exclusively top-view particles. A Tecnai Polara (FEI) electron microscope working at 300 KV with a magnification of 41,322 was used to automatically collect images with a K2-summit direct electron detector camera in counting mode using the Latitude S software (Gatan Inc). Movie beam induced motion correction was performed with the motioncor2 software [51] and CTF estimation with Gctf [52]. The pixel size at the detector level was 1.21 Å. For each sample, semi-automated particle picking with EMAN boxer [53] was performed followed by 2D image classification by using Relion [32] on ~5,000 manually boxed particles. With the obtained 2D class averages (S3 Fig) a low-resolution *ab-initio* 3D model was obtained by using RIco [54] (S6 Fig). 3D Image processing then followed the standard Relion protocol [32] with the need of a 3D classification step to reduce the excess of top-views. C15 symmetry was imposed.

From a total of 2,021 micrographs of EpsD, 46,126 particles were kept in the final 3D reconstruction at 3.4 Å resolution (FSC 0.143). For ExeD, 2,247 micrographs were collected and 51,960 particles were kept in the final 3D reconstruction at 3.7 Å resolution (FSC 0.143). Model building was performed using Buccaneer [55] and Coot [56] based on secretins with PDB codes 5WQ8 and 5WQ7 for EpsD and ExeD, respectively. Refinement was performed with Refmac [57].

### Bacterial strains, growth conditions, and immunoblots

*Vibrio vulnificus* strain 67181283 and mutants thereof where routinely grown in Luria-Bertani (LB) or AKI media [58] at 30 °C until the mid to late logarithmic growth phase, while *A*. *hydrophila* strain Ah65 and the *ΔepsD* mutant AhD14 were grown in buffered LB [24]. Antibiotics were used at the following concentrations: rifampicin, 50 μg/mL; ampicillin, 100 μg/mL, chloramphenicol, 1 μg/mL. pMMB-TS166 was constructed by nested PCR amplification of the *epsS* ORF of *V*. *vulnificus* with oligonucleotides US887/US889 in the first amplification, US888/US890 in the second. The resultant amplicon was cloned into pMMB67EH [59] with the NEBuilder HiFi DNA assembly reaction. pExeD-EpsD-α-12 was constructed by amplifying an upstream fragment of *exeD* with oligonucleotides US952 and US976 and the α-12 encoding region of *epsD* with oligonucleotides US983 and US984 followed by cloning of both amplicons into *SalI* and *HindIII* -cleaved pVACD using the HiFi DNA assembly reaction. Similar methods were used employing the primer pairs US952/US976, US952/US977 and US952/US978 to create pExeD-EpsD-α-12E, US952/US979 and US980/US981 to create pExeD-EpsD-S, and US952/US985 to create pExeD-ΔS. All clones were confirmed by sequencing. The multimeric and monomeric forms of EpsD and ExeD were identified by immunoblot analysis of whole-cell samples from liquid culture as described [31]. EpsS antibodies were generated in New Zealand White rabbits using N-terminal His-tagged EpsS.

### Construction of *eps* chromosomal mutants

A kanamycin resistance cassette insertion mutation of *epsA* in *V*. *vulnificus* was previously described [31]. An in-frame deletion mutation of the *epsS* open-reading frame was constructed by allelic exchange employing the counter-selectable suicide vector pWM91. Approximately 1,000 bp upstream (including the *epsS* start codon) and 1,000 bp downstream (including the *epsS* stop codon) were amplified by primer pairs US868/US869 and US870/US871, respectively. pWM91 was linearized with XhoI and the 3 fragments were assembled with the NEBuilder HiFi DNA assembly reaction (New England Biolabs). The deletion vector was transformed into SM10λpir and conjugated into WT and the *exeA* deletion mutant of *V*. *vulnificus*. Cointegrants were selected by growth on rifampicin (50 μg/mL) and ampicillin (100 μg/mL) at 30 °C. Transconjugants were grown in LB for 6 h then plated onto LB containing 6% sucrose for counter-selection. Sucrose resistant colonies were screened for ampicillin sensitivity and *epsS* deletion mutants were confirmed by PCR with primers US872/US873 and immunoblotting with an anti-EpsS antibody.

### Membrane fractionation, vesicle isolation and secretion analysis

Inner and outer membranes were fractionated from culture lysates essentially as previously described [21], except that the cultures were grown to late-log phase in buffered LB, and the cells were ruptured by two passages through a French pressure cell operating at 16,000 psi. Membrane fractions were assayed for NADH oxidase activity to identify inner membrane vesicles and were electrophoresed on 4–15% acrylamide gels to identify outer membrane vesicles via the presence of the major porin protein II [24]. Vesicles were isolated from unconcentrated culture supernatants by filtration through a 0.2 μm filter followed by ultracentrifugation at 150,000 x g for 3 h followed by resuspension in 1/25 of the original volume of LB. Lipase activities in culture supernatants were measured by following the release of *p*-nitrophenol from *p*-nitrophenol caprylate (28), while protease activity was measured using resorufin-labelled casein (Roche) according to the manufacturer’s instructions. Aerolysin titres were determined via 2-fold serial dilution of culture supernatants in a microtitre plate assay utilizing a 0.8% solution of mouse erythrocytes [23].

### EpsS crystallization, data collection, and structure solution

Initial crystallization conditions for purified EpsS were obtained by the HTX platform (ISBG, Grenoble, France). Subsequently, urchin-like crystals were improved manually, and improved crystals were grown using the vapor diffusion method in a hanging drop setup by mixing 1 μl of EpsS (in 25 mM HEPES pH 7.5, 150 mM NaCl at 5.5 mg/ml) with 1 μl of 0.1 M Tris-HCl pH 7.5, 1.9 M Ammonium Sulfate, 2.5% (v/v) PEG 400. After one month of growth at 20 °C, crystals were fished and soaked in the crystallization solution supplemented with 25% (v/v) ethylene glycol and then flash cooled with a cryo-loop in liquid nitrogen. X-ray data collection was performed under a cold stream of nitrogen at 100 K at the European Synchrotron Radiation Facility (ESRF, Grenoble, France) on beamline ID30A-3. Data were recorded to 1.6 Å on an Eiger X 4M detector.

X-ray diffraction data were analyzed, indexed and scaled with XDS [60] and then imported into the CCP4 program suite [61]. Crystals belong to space group I222; due to the high resolution of the data set, we employed Arcimboldo Schredder [44] to solve the structure using AspS as an initial model (PDB: 4FTF). Arcimboldo Schredder was run in the laboratory’s Linux OpenSuSE HTCondor v8.6.6 cluster [62] with 68 CPUs. ARP/wARP [45] was used to rebuild the solved model and reduce bias. COOT [56] and REFMAC5 [57] were subsequently employed to manually build and refine EpsS. TLS definition was introduced at the later REFMAC refinements step [63]. The quality of EpsS model was determined with MolProbity [64] and PROCHECK [65]. Figures were generated with PyMol (http://www.pymol.org).

## Supporting information

S1 Fig Purification of *A*. *hydrophila* ExeD and *V*. *vulnificus* EpsD multimers following *in vitro* synthesis. **(A)** The soluble fraction (S) of the ExeD *in vitro* synthesis reaction was separated from liposomes (L). Following detergent extraction of the liposomes, the preparation was fractionated by Ni-NTA column chromatography. Fractions 26–29 were pooled (AS) and fractionated by Superose 6 column chromatography **(B)** resulting in separation of ExeD multimers (fraction 10) from residual monomers (fraction 17). **(C,D)** Similar *in vitro* synthesis and Ni-NTA fractionation of EpsD allowed final Superose 6 purification of multimers (Fraction 15).
(TIF)

S2 Fig Examples of cryo-EM micrographs of ExeD. The bottom image was taken using a Quantifoil holey carbon grid with an extra carbon layer in order to increase the number of ExeD molecules with side views. The scale bar corresponds to 500 Å.
(TIF)

S3 Fig Initial 2D class averages from manually picked EpsD particles. (TIF)

S4 Fig Fourier-shell correlation for ExeD (top) and EpsD (bottom) indicating the estimated resolution for the presented 3D reconstructions. The red line is the FSC with randomized phases, the green line is the unmasked FSC, the blue line is the masked FSC and the black line is the FSC corrected for the masking effect [66].
(TIF)

S5 Fig Comparison of crystal structures of EpsS and AspS, the latter from the cryo-EM structure of the GspD:AspS complex. Both proteins display a central β-sheet surrounded by α-helices. The region involving β-1 and all interstrand interconnecting loops, traceable in our high resolution structure of EpsS, is absent in the structure of AspS obtained from the cryo-EM map of GspD [41].
(TIF)

S6 Fig *Ab-initio* 3D initial model obtained from the 2D class averages of EpsD. The model is shown in two different orientations.
(TIF)

S1 Table Cryo-electron microscopy data collection and refinement statistics. (DOCX)

S2 Table X-ray crystallography data collection and refinement statistics. (DOCX)

S3 Table Oligonucleotides used in this study. (DOCX)
