# Supplementary figures and images for: Structure and assembly of pilotin-dependent and -independent secretins of the type II secretion system
Source: PLoS Pathog. 2019 May 13;15(5):e1007731. doi: 10.1371/journal.ppat.1007731 (PMC6532946; doi:10.1371/journal.ppat.1007731)

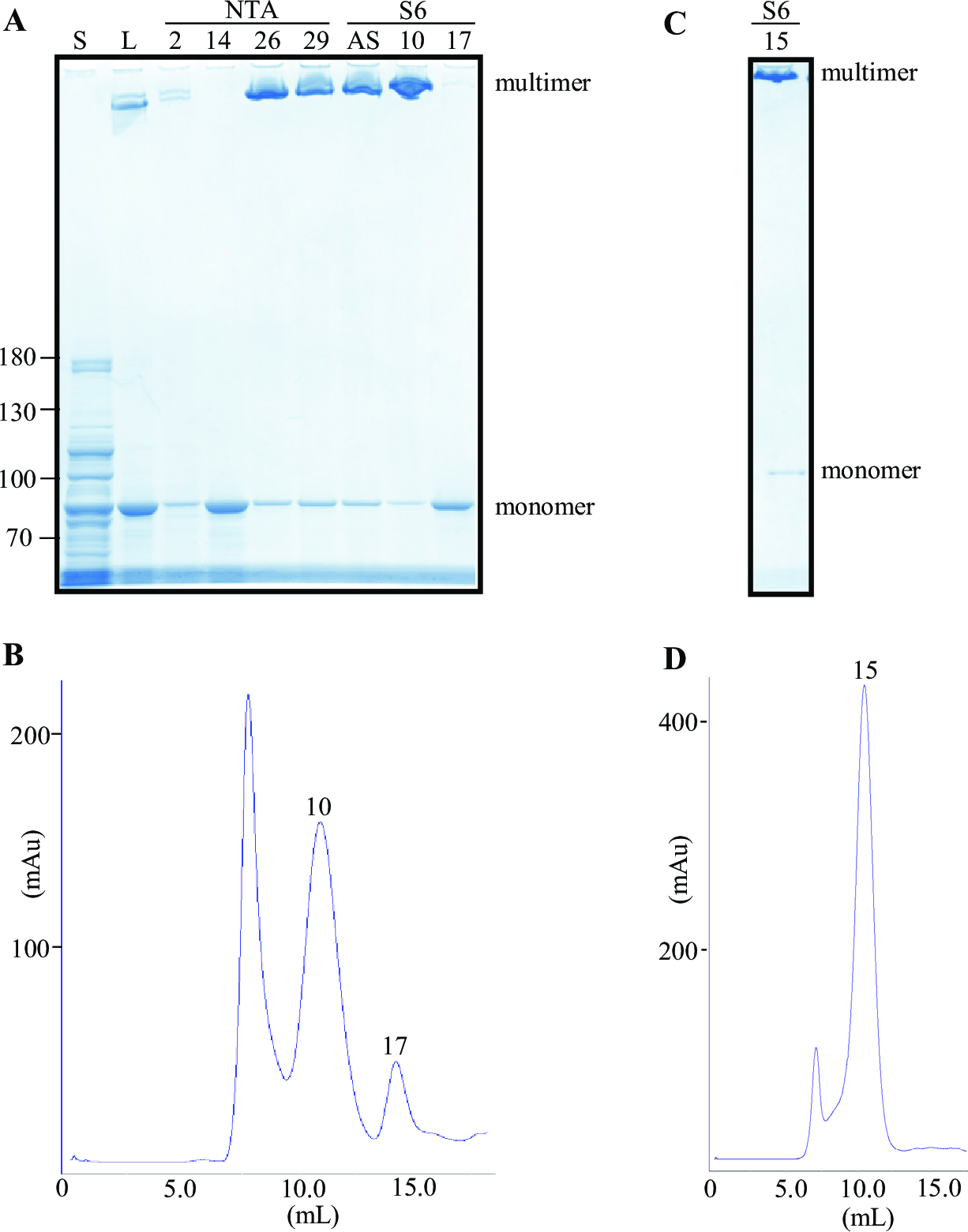

Supplement: S1 Fig — (A) The soluble fraction (S) of the ExeD in vitro synthesis reaction was separated from liposomes (L). Following detergent extraction of the liposomes, the preparation was fractionated by Ni-NTA column chromatography. Fractions 26–29 were pooled (AS) and fractionated by Superose 6 column chromatography (B) resulting in separation of ExeD multimers (fraction 10) from residual monomers (fraction 17). (C,D) Similar in vitro synthesis and Ni-NTA fractionation of EpsD allowed final Superose 6 purification of multimers (Fraction 15). (TIF) [file ppat.1007731.s001.tif]

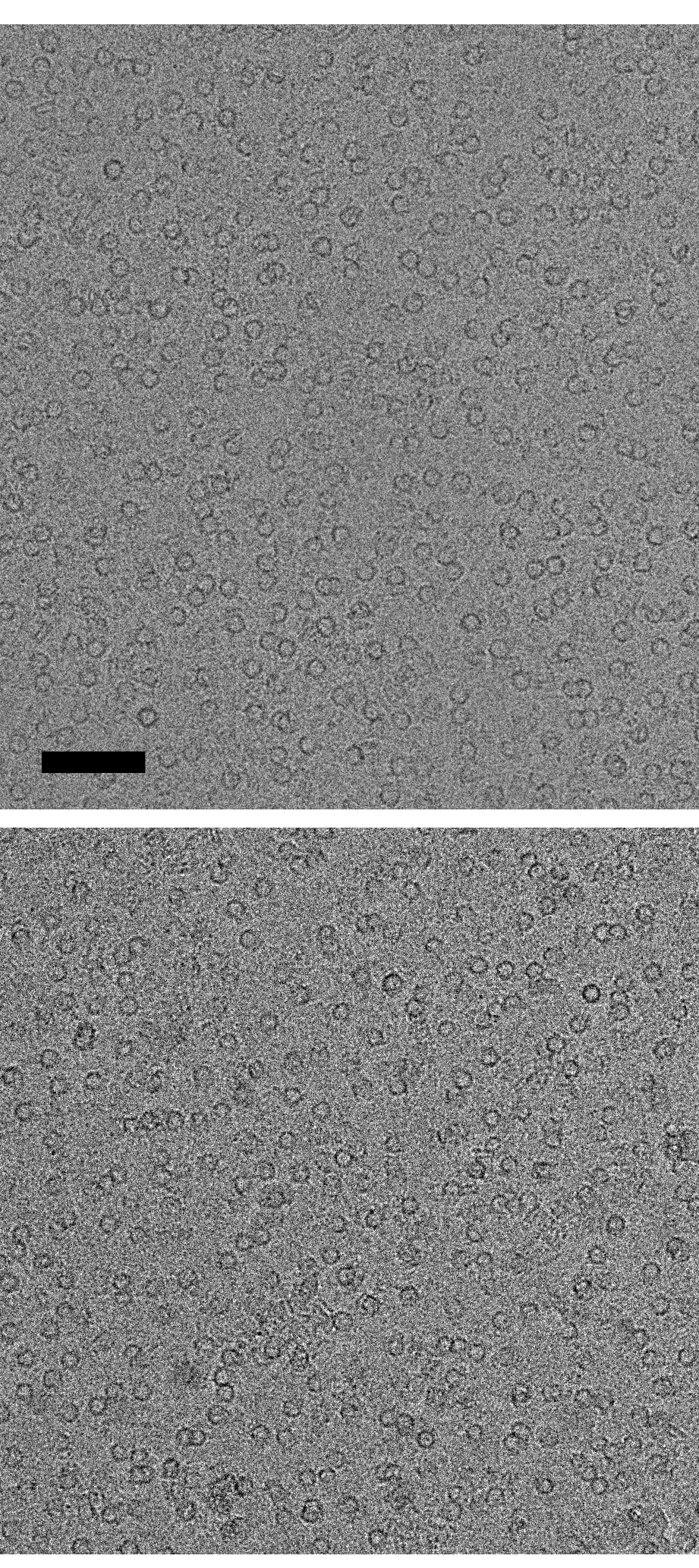

Supplement: S2 Fig — The bottom image was taken using a Quantifoil holey carbon grid with an extra carbon layer in order to increase the number of ExeD molecules with side views. The scale bar corresponds to 500 Å. (TIF) [file ppat.1007731.s002.tif]

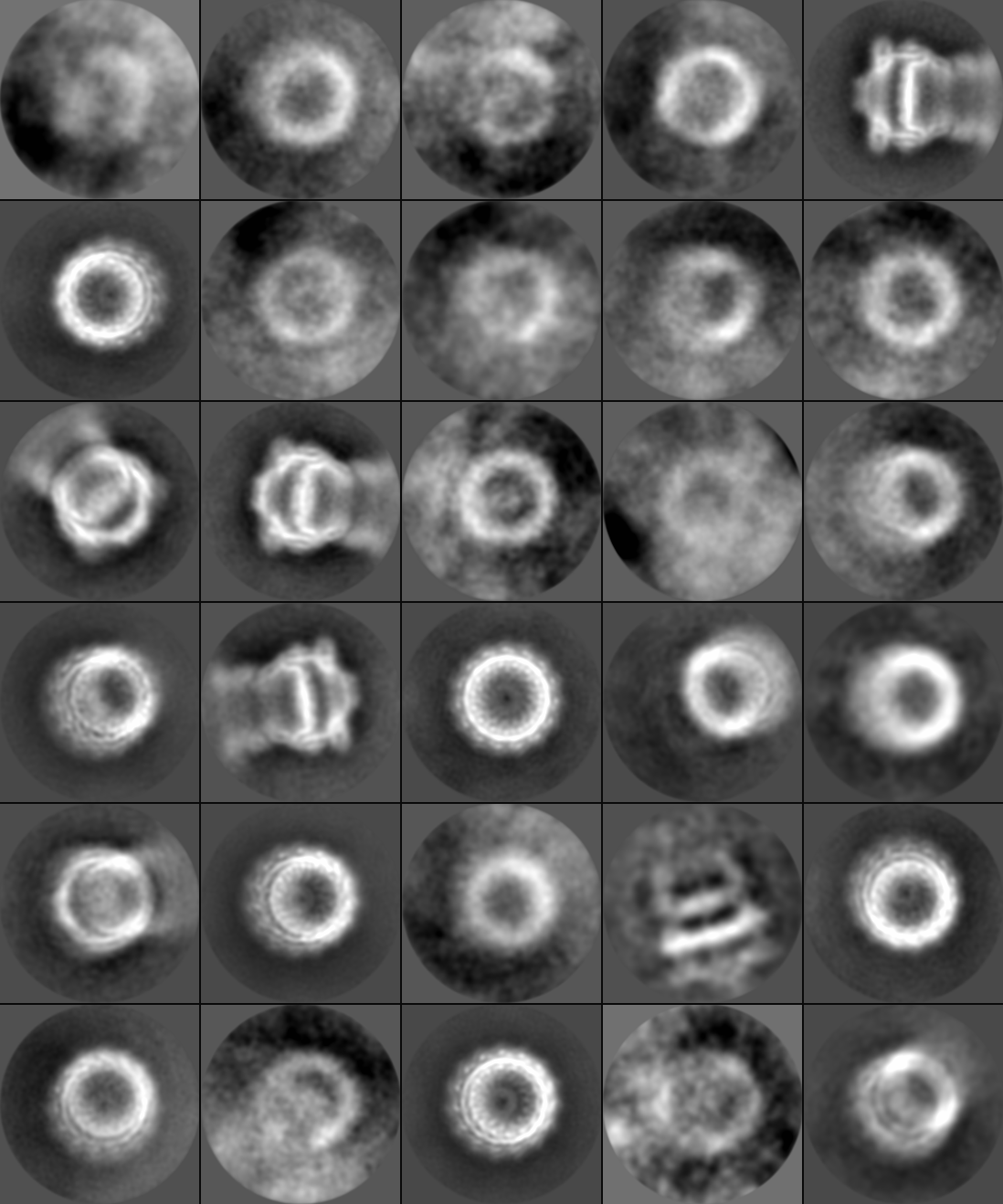

Supplement: S3 Fig — (TIF) [file ppat.1007731.s003.tif]

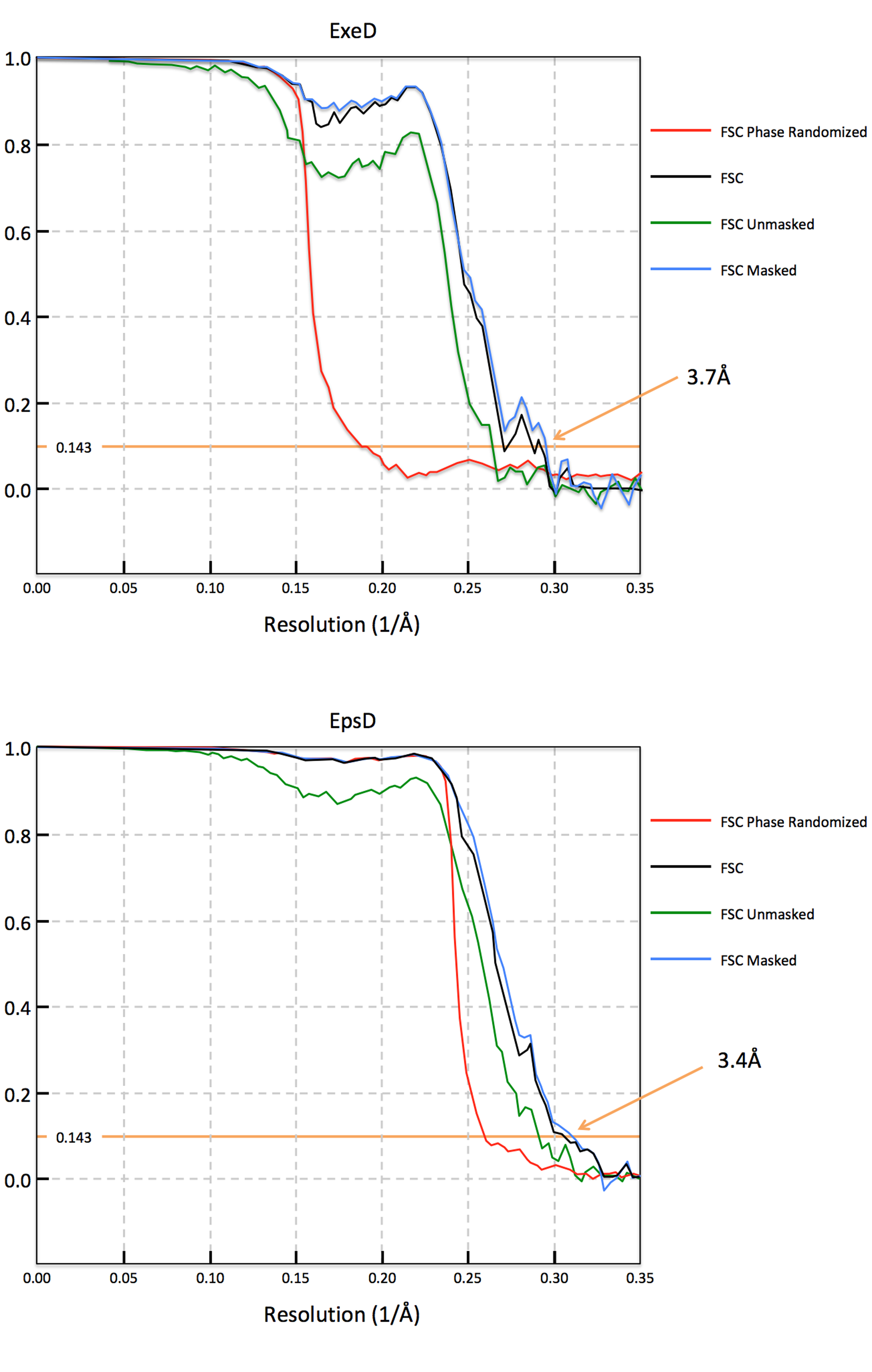

Supplement: S4 Fig — The red line is the FSC with randomized phases, the green line is the unmasked FSC, the blue line is the masked FSC and the black line is the FSC corrected for the masking effect [66]. (TIF) [file ppat.1007731.s004.tif]

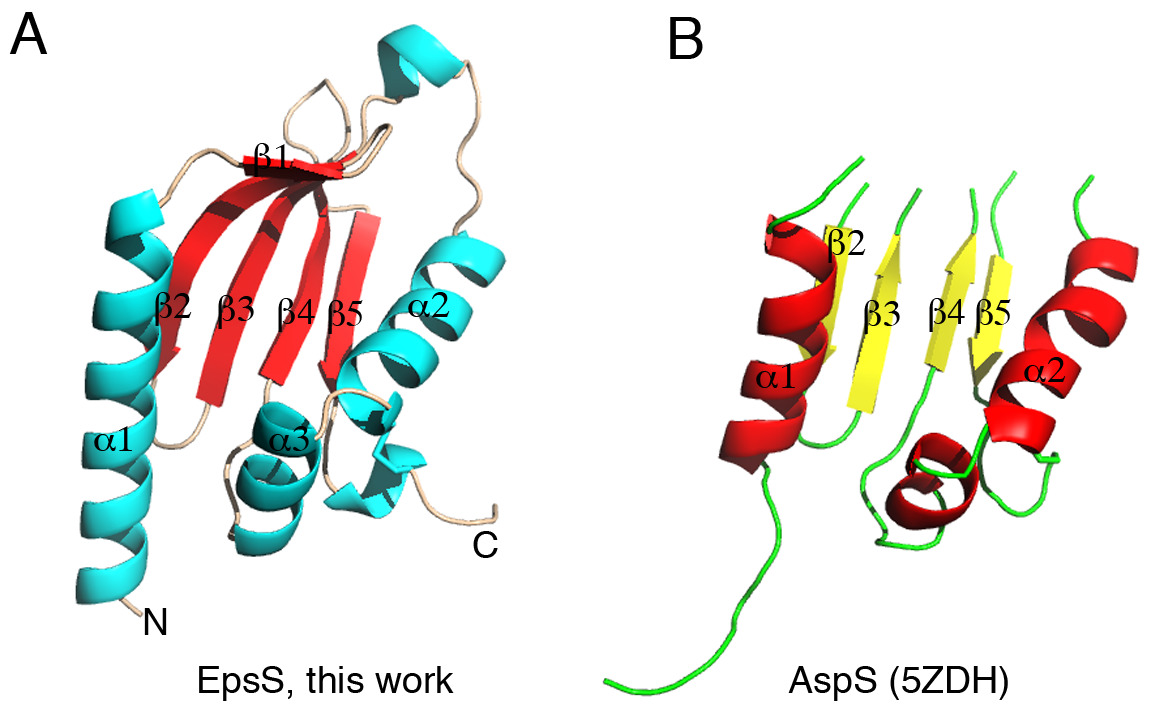

Supplement: S5 Fig — Both proteins display a central β-sheet surrounded by α-helices. The region involving β-1 and all interstrand interconnecting loops, traceable in our high resolution structure of EpsS, is absent in the structure of AspS obtained from the cryo-EM map of GspD [41]. (TIF) [file ppat.1007731.s005.tif]

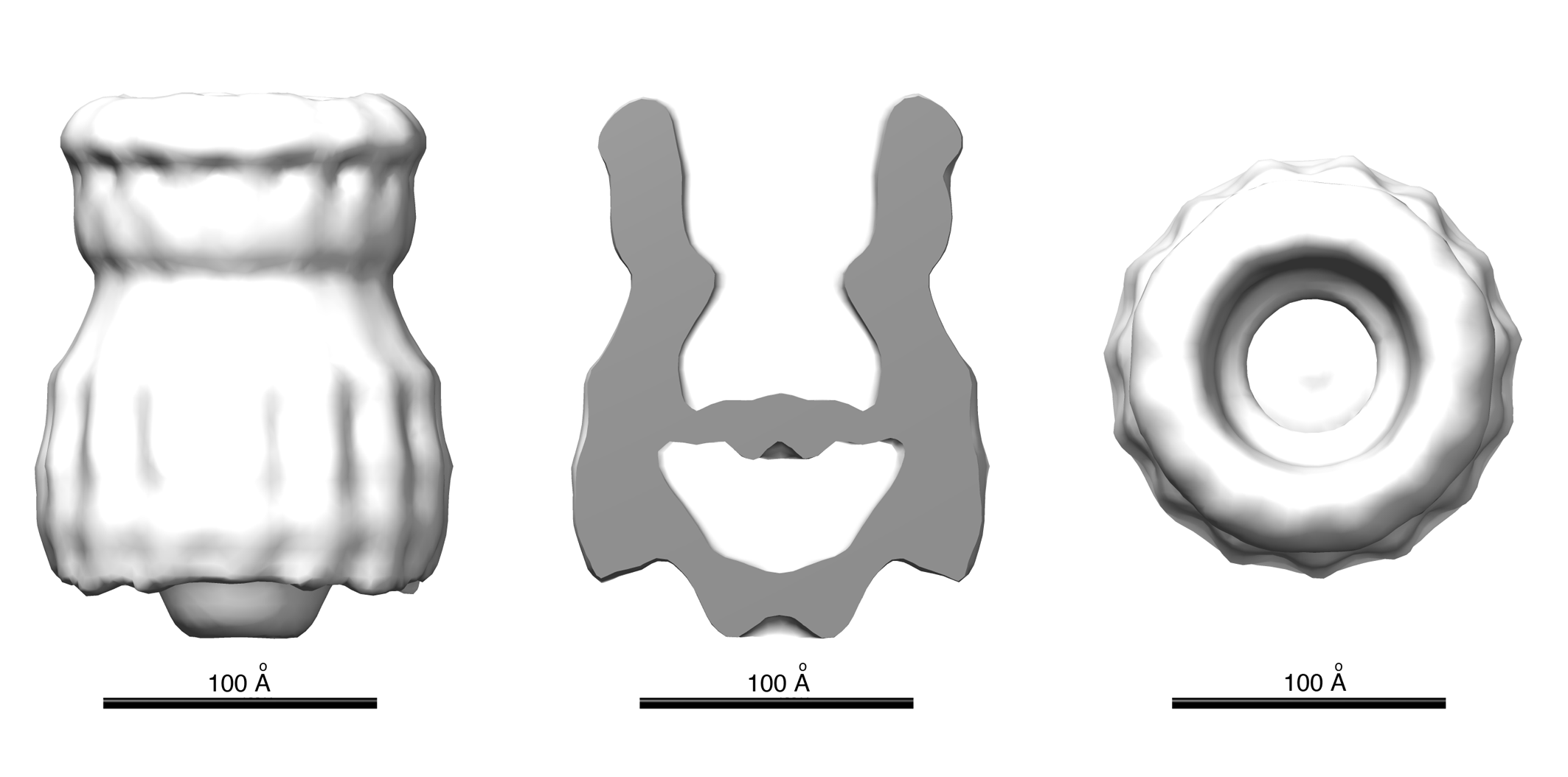

Supplement: S6 Fig — The model is shown in two different orientations. (TIF) [file ppat.1007731.s006.tif]
