# Supplementary material for: Structure and assembly of pilotin-dependent and -independent secretins of the type II secretion system
Source: PLoS Pathog. 2019 May 13;15(5):e1007731. doi: 10.1371/journal.ppat.1007731 (PMC6532946; doi:10.1371/journal.ppat.1007731)
Supplement: S1 Table — (DOCX) [file ppat.1007731.s007.docx]

**Table S1: Cryo-electron microscopy data collection and refinement statistics**

|  | EpsD | ExeD |
| --- | --- | --- |
| Voltage (kV) | 300 | 300 |
| Pixel size (Å) | 1.21 | 1.21 |
| Electron exposure (e−/ Å2) | 40 | 40 |
| Defocus range (μm) | 0.7-4.2 | 0.5-4.6 |
| Symmetry imposed | C15 | C15 |
| Initial particle images | 125884 | 272432 |
| Final particle images | 46126 | 51982 |
| Resolution (Å) | 3.4 | 3.7 |
| FSC threshold | 0.143 | 0.143 |
| Map-sharpening *B* factor (Å2) | -146.221272 | -182.614233 |
