# Supplementary material for: Structure and assembly of pilotin-dependent and -independent secretins of the type II secretion system
Source: PLoS Pathog. 2019 May 13;15(5):e1007731. doi: 10.1371/journal.ppat.1007731 (PMC6532946; doi:10.1371/journal.ppat.1007731)
Supplement: S2 Table — (DOCX) [file ppat.1007731.s008.docx]

**Table S2: X-ray crystallography data collection and refinement statistics**

| X-ray source | ID30-A3 |
| --- | --- |
| Detector | Eiger 4M |
| Wavelength (Å) | 0.9677 |
| Scan range (°) | 450 |
| Oscillation (°) | 0.3 |
| Space group | I222 |
| *a* (Å) | 35.83 |
| *b* (Å) | 83.21 |
| *c* (Å) | 90.79 |
| Overall Resolution (Å) | 45.39-1.75 |
| No. Observed/Unique reflection | 223,882 /14,037 |
| High resolution shell (Å) | 1.85-1.75 |
| Completeness (%) (last shell) | 99.3 (95.8) |
| R sym (last shell) | 13 (115) |
| I/s(I) (last shell) | 14.14 (2.12) |
| CC1/2 (last shell) | 99.9 (88.0) |
| Wilson plot B-factor (Å) | 28.85 |
| *Arcimboldo/ Shredder* |  |
| Molecule/ASU | 1 |
| LLG score | 164 |
| *Refinement* |  |
| Rwork/Rfree (%) | 0.179/0.228 |
| RMS deviation, bond lengths (Å) | 0.012 |
| RMS deviation, bond angles (°) | 1.643 |
| Mean B-factor (Å²) | 25.78 |
| No. of protein atoms | 1059 |
| No. of water molecules | 94 |
| Residues in most favoured/allowed region of Ramachandran plot (%) | 100 |
