# Supplementary material for: Structure and assembly of pilotin-dependent and -independent secretins of the type II secretion system
Source: PLoS Pathog. 2019 May 13;15(5):e1007731. doi: 10.1371/journal.ppat.1007731 (PMC6532946; doi:10.1371/journal.ppat.1007731)
Supplement: S3 Table — (DOCX) [file ppat.1007731.s009.docx]

**Table S3: Oligonucleotides used in this study**

Name Sequence

VvD24TF ACAACAGGATCCAACGAGTTTAGTGCAAGCTT

VvD24TR TGTTGTCTCGAGTTACTGCTGTTCCATTTGATCG

AhD24TF ACAACAGGATCCACCGAGTATTCTGCCAGCTTCA

AhD24TR TGTTGTCTCGAGTTACTTGTTACCCTGAACGAATGG

EpsSTEVF CTGCTGTATTTTCAGGGCTCATCTTCAAATGGTGAGAAAGAG

EpsSTEVR TCGACGTAGGCCTTTCTAGGTTAATTAAGCCTCGAGCTA

US868 GAATTGGGTACCGGGCCCCCCCTGTTGGGCAAAGAGATCACC

US869 CTGACAACCCATGGTCACTACACTAACGATGCAGAGAGTAC

US870 GTTAGTGTAGTGACCATGGGTTGTCAGCCACAATAGCGG

US871 AAGCTTATCGATACCGTCGACCACGTAATTCAGCCTGTGACGG

US872 ATTGCAGCAACAGCCATTCAG

US873 CATTCAAATGCTTGAAGGCGAC

US887 AGGAAACAGAGGAGGTGTGTTCTAATG

US888 ATAACAATTTCACACAGGAAACAGAGG

US889 CGACTCTAGACTATTGTGGCTGACAAC

US890 TGCATGCCTGCAGGTCGACTCTAGACTA

US952 GGTGGAAGTGGTTCGCCGGGTCGACAAGGCAGGGGATCAGGAAG

US976 GATAAAGGCTTGAATTTCAGCCGGCATGGTCACATCCTGGCCATA

US977 TTACTGCTGTTCCATTTGATCGATAAAGGCTTGAATTTCAGCCGG

US978 TCTCATCCGCCAAAACAGCCAAGCTTTTACTGCTGTTCCATTTG

US979 GTAATACCATCCGCCGTCATACCATCCCGCAATATGGTGGGCCGG

US980 GGCCCACCATATTGCGGGATGGTATGACGGCGGATGGTATTAC

US981 TCTCATCCGCCAAAACAGCCAAGCTTTTACTGCTGTTCCATTTGA

US983 CAAGCCTTTATCGATCAAATGAAAACACACCAGCAGGCG

US984 TCTCATCCGCCAAAACAGCCAAGCTTGATTCGTGTCATCAA

US985 TCTCATCCGCCAAAACAGCCAAGCTTTCAGTTTGCATCCCGCAAT
